# Association between gastrointestinal tract infections and glycated hemoglobin in school children of poor neighborhoods in Port Elizabeth, South Africa

**DOI:** 10.1371/journal.pntd.0006332

**Published:** 2018-03-15

**Authors:** Nan Shwe Nwe Htun, Peter Odermatt, Ivan Müller, Peiling Yap, Peter Steinmann, Christian Schindler, Markus Gerber, Rosa Du Randt, Cheryl Walter, Uwe Pühse, Jürg Utzinger, Nicole Probst-Hensch

**Affiliations:** 1 Department of Epidemiology and Public Health, Swiss Tropical and Public Health Institute, Basel, Switzerland; 2 University of Basel, Basel, Switzerland; 3 Department of Sport, Exercise and Health, University of Basel, Basel, Switzerland; 4 Institute of Infectious Disease and Epidemiology, Tan Tock Seng Hospital, Singapore, Singapore; 5 Department of Human Movement Science, Nelson Mandela University, Port Elizabeth, South Africa; London School of Hygiene and Tropical Medicine, UNITED KINGDOM

## Abstract

**Background:**

Low- and middle-income countries are facing a dual disease burden with infectious diseases (e.g., gastrointestinal tract infections) and non-communicable diseases (e.g., diabetes) being common. For instance, chronic parasite infections lead to altered immune regulatory networks, anemia, malnutrition, and diarrhea with an associated shift in the gut microbiome. These can all be pathways of potential relevance for insulin resistance and diabetes. The aim of this study was to investigate the association between common gastrointestinal tract infections and glycemia in children from non-fee paying schools in South Africa.

**Methodology:**

We conducted a cross-sectional survey among 9- to 14-year-old school children in Port Elizabeth. Stool and urine samples were collected to assess infection status with parasitic worms (e.g., *Ascaris lumbricoides*, *Enterobius vermicularis*, and *Trichuris trichiura*), intestinal protozoa (e.g., *Cryptosporidium parvum* and *Giardia intestinalis*), and the bacterium *Helicobacter pylori*. Glycated hemoglobin (HbA1c) was measured in finger prick derived capillary blood. All children at schools with a high prevalence of helminth infections and only infected children at the schools with low infection rates were treated with albendazole. The association of anthelmintic treatment with changes in HbA1c 6 months after the drug intervention was also investigated.

**Findings:**

A high prevalence of 71.8% of prediabetes was measured in this group of children, with only 27.8% having HbA1c in the normal range. *H. pylori* was the predominant infectious agent and showed an independent positive association with HbA1c in a multivariable regression analysis (β = 0.040, 95% confidence interval (CI) 0.006–0.073, p<0.05). No association of HbA1c with either any other infectious agent or albendazole administration was found.

**Conclusion:**

The role of *H*. *pylori* in diabetes needs confirmation in the context of longitudinal treatment interventions. The specific effect of other gastrointestinal tract infections on glycemia remains unclear. Future studies should integrate the measurement of biomarkers, including immunological parameters, to shed light on the potential mediating mechanisms between parasite infections and diabetes.

## Author summary

Parasitic worms (e.g., pinworm, roundworm, and whipworm), intestinal protozoa (e.g., *Cryptosporidium parvum* and *Giardia intestinalis*), and the bacterium *Helicobacter pylori* persist at high rates in the gastrointestinal tract of people from low- and middle-income countries. These infectious agents are increasingly paralleled by high rates of non-communicable diseases, such as diabetes. We studied the association of glycemia, measured as HbA1c with common gastrointestinal tract infections among school children aged 9–14 years from disadvantaged neighborhoods in Port Elizabeth, South Africa. Our goal was to deepen the understanding of whether specific gastrointestinal tract infections might be early life determinants of elevated HbA1c levels that might lead to diabetes. We found that the bacterium *H*. *pylori* was very common among our group of children with a positive association with hyperglycemia. None of the other infectious agents showed such an association. Additional, longitudinal studies are needed to determine whether there is causality for the observed association between *H*. *pylori* and hyperglycemia. The integration of biomarkers will allow studying mediating mechanisms.

## Introduction

In low- and middle-income countries (LMICs), the dual disease burden stemming from infectious diseases (IDs) and non-communicable diseases (NCDs) poses a challenge to population health and the health systems. Soil-transmitted helminths and schistosomes are estimated to infect over a billion individuals in LMICs [1, 2] and cause abdominal pain, diarrhea, poor cognitive development, malnutrition, and anemia. As a consequence of such symptoms, school and work performance is affected and physical activity levels compromised [3]. Helminthiases are often chronic, a result of both under-treatment and re-infection. Soil-transmitted helminthiasis, schistosomiasis, and intestinal protozoa infection are intimately connected with poverty, partially explained by lack of clean water, sanitation, and hygiene [4].

NCDs are gaining importance, also in LMICs [5]. For example, the frequency of diabetes mellitus (DM) is rising worldwide, and South Africa is among the top five countries in Africa with an estimated DM prevalence of 9.2% [6]. This can be attributed primarily to aging, population growth, increasing rates of unhealthy dietary habits, a sedentary lifestyle, and obesity. While NCDs and DM particularly affect older people, it is generally accepted that early life exposures contribute to the accumulation of molecular damage and a higher disease risk later in adulthood [7].

Little is known about how common parasite infections affect glucose homeostasis and DM etiology, particularly at young age. It is conceivable that parasite infections influence the DM risk through different pathways and in opposite directions [8]. On the one hand, parasite-induced alterations of immune regulatory networks, which have evolved to prolong survival in the human intestines, may stimulate anti-inflammatory pathways and decrease the risk of obesity-induced insulin resistance. Malnutrition, diarrhea and, as a result, low body weight related to chronic helminth infections may additionally decrease DM risk. On the other hand, a sedentary lifestyle and anemia have the potential to increase DM risk. Additionally, the mediating role of helminth-induced shifts in the gut microbiome composition remains to be determined [9].

A limited number of recently reviewed epidemiologic studies with inconsistent results investigated the cross-sectional association of different IDs, including lymphatic filariasis [10, 11], schistosomiasis [12], strongyloidiasis [13, 14], and soil-transmitted helminthiasis [15, 16] with DM or insulin sensitivity. In the present study, we followed up on these observations by studying the association of gastrointestinal tract infections due to helminths, intestinal protozoa, and the bacterium *Helicobacter pylori* with glycated hemoglobin (HbA1c) concentration in school children in the frame of the “Disease, Activity and Schoolchildren’s Health” (DASH) study in Port Elizabeth, South Africa [3, 17]. The study provided detailed information on physical activity, fitness, and socioeconomic status (SES) to consider as confounding factors on gastrointestinal tract infection status, and intensity of helminth infections to study a possible dose-response relationship; and on the longitudinal course of HbA1c upon selective anthelmintic treatment.

## Methods

### Ethics statement

Ethics approval was obtained from ethics committees in both Switzerland (EKNZ; reference no. 2014–179, approval date: 17 June 2014), and South Africa (study number H14-HEA-HMS002, approval date: 4 July 2014). Written informed consent from the parents/guardians of participating children as well as oral assent from the children were obtained prior to data collection.

### Study setting and design

A total of 1,009 grade-4 children aged 9–14 years from eight non-fee paying primary schools were recruited in various parts of Port Elizabeth in the south-eastern part of South Africa, as described before [3, 17]. The study was part of the 2-year longitudinal DASH study that consisted of three cross-sectional surveys. In each of the cross-sectional surveys, children’s gastrointestinal infections and other health parameters were assessed, including HbA1c, anthropometry, levels of physical fitness, cognitive performance, and psychosocial health. After each survey, helminth-infected children were treated with a single 400 mg oral dose of albendazole. In schools where the prevalence of helminth infection was 20% or above, all children were treated regardless of infection status according to guidelines put forth by the World Health Organization (WHO) [18]. Children with other infections (*Cryptosporidium* spp. and/or *Giardia intestinalis*) in combination with severe symptoms (e.g., bloody stool, diarrhea, abdominal pain, and any abnormal lung sounds) were referred to the nearest local health clinic for individual management.

The baseline cross-sectional survey took place in March 2015. The current study considers data from this baseline survey and the anthelmintic treatment follow-up examination in September/October 2015.

### Inclusion and exclusion criteria

Grade-4 primary school children of the selected schools were included in the study. Children with severe clinical signs and symptoms (e.g., severe fever, severe headache, dizziness, nausea, skin rashes, seizures, and diarrhea) or reported serious health problems, such as Crohn’s disease, liver or kidney diseases, or who participated in any other study were excluded.

### Procedures

#### Questionnaires and interviews

Standardized questionnaires available in both English and local languages (Afrikaans and Xhosa) were used to determine the SES of the children and their families. Volunteers fluent in relevant languages were trained to conduct these in-person interviews.

#### Clinical and anthropometric assessment

Experienced nurses obtained a detailed medical history through physical examination of the whole body and evaluation of symptoms to assess current infections, anemia, jaundice, as well as signs and symptoms of protein energy malnutrition, general respiratory and gastrointestinal problems, allergies, and skin infections. Body temperature was measured using an infrared digital ear thermometer (TS7, Hi-Care International; Cape Town, South Africa). Blood pressure was measured once after the child had been seated for 5 min using validated oscillometric Omron digital blood pressure monitor (Omron M6 AC model; Hoofdoorp, Netherlands).

For the anthropometric measurements, shoes and sweaters were removed before standing on a digital weighting scale (Micro T7E electronic platform scale, Optima Electronics; George, South Africa). Body weight was measured once to the nearest 0.1 kg. Children’s height was assessed with a Seca stadiometer (Surgical SA; Johannesburg, South Africa), whereby the child was standing with the back erect, heals touching the wall, and shoulders relaxed. Body height was taken to the nearest 0.1 cm.

#### Stool and urine sampling for assessment of gastrointestinal tract infection

A sample of at least 15 g of early morning stool from every participant was collected in a container and transferred to a laboratory of the Nelson Mandela University (NMU) in Port Elizabeth for diagnostic work-up. Stool samples were visually examined for *Taenia* spp. proglottids, signs of blood, mucus, and diarrhea. Duplicate 41.7 mg Kato-Katz thick smears were prepared from each stool sample [19] and examined under a microscope by two experienced laboratory technicians. The number of helminth eggs was counted and recorded for each species separately. Helminth egg counts were multiplied by a factor of 24 to obtain a proxy for infection intensity, expressed as the number of eggs per gram of stool (EPG), which was then categorized into light, moderate, and heavy infections using readily available cut-offs offered by WHO [20]. For the detection of intestinal protozoa *C*. *parvum* and *G*. *intestinalis*, a Crypto-Giardia Duo-Strip rapid diagnostic test (RDT) was performed on the stool sample [21]. For the discovery of the bacterium *H*. *pylori*, a Pylori-Strip RDT was applied [22] (both tests are from CORIS, BioConcept; Gembloux, Belgium).

Children were also asked to provide a urine sample, which was transferred to the laboratory and analyzed on the same day. Visual inspection for macrohematuria was followed by testing for blood in urine using Hemastix strips (Siemens Healthcare Diagnostics GmbH; Eschborn, Germany), as a proxy for *Schistosoma haematobium* infection. A point-of-care circulating cathodic antigen (POC-CCA) urine cassette test (Rapid Medical Diagnostics; Cape Town, South Africa) was used for the diagnosis of *Schistosoma mansoni* infection [23].

The infectious agents under the same taxonomy were grouped as trematodes (*S*. *mansoni* and *S*. *haematobium*), nematodes (*Ascaris lumbricoides*, *Enterobius vermicularis*, and *Trichuris trichiura*), intestinal protozoa (*G*. *intestinalis* and *C*. *parvum*), and bacteria (*H*. *pylori*) in the statistical analyses.

#### HbA1c measurement

HbA1c reflects plasma glucose concentrations over an 8- to 12-week period. It is used as a convenient diagnostic indicator for DM, as no fasting is required to measure it. HbA1c concentrations were obtained by using the POC instrument Afinion (Alere Inc. Waltham; Waltham, MA, USA), which is based on boronate affinity separation and the use of fluorescence quenching, with results available after 3 min. This method meets the generally accepted performance criteria for HbA1c, as defined by the U.S. National Glycohemoglobin Standardization Program (NGSP), with no interference from HbC, HbS, HbE, and HbD traits results. All test cartridges for the Afinion test belonged to the specific lot number. Test cartridges were stored at 4°C during the study and were removed from the refrigerator a maximum of 120 min before the test. The tests were run when the temperature of the cartridges were in their optimal range (15–25°C). Ambient room temperature was measured on each test day to assure absence of temperature effects on HbA1c test results as a means of quality control. Patients with HbA1c ≥6.5%, the recommended cut-off for diagnosing DM [24], were referred to DM care centers for confirmation and specific management.

#### Hemoglobin (Hb) measurement

Hb concentration was measured with the HemoCue Hb 301 system (HemoCueAB; Ängelholm, Sweden) and the results were considered to the nearest 0.1 g/l.

### Covariates information

The SES was derived from housing characteristics and household assets (S1 Table). The SES score of the households was categorized as poorest, poor, and least poor using the scale by Filmer and Pritchett to disaggregate the distribution of the scores [25].

The age of individuals was grouped into five categories (8–9, 10, 11, 12 and >12 years), according to the age distribution of the population in the study. The body mass index (BMI) was calculated as kg/m^2^ based on the measured height and weight. For physical activity, we used questionnaires on the frequency and duration of certain activities (how many days in a week the children were physically active for a total of at least 60 min, the traveling time from home to school, and numbers of exercising days and intensity of exercise in children’s leisure time). The scores were summed up and equally categorized into tertiles: active, fair, and poor physical activity level according to the distribution of scores.

Cardiorespiratory fitness (VO_2_ max) is the maximum rate of oxygen consumption, as measured during incremental exercise. We estimated the individual VO_2_ max from the 20 m shuttle run test, which is the most widely used field test for determining cardiorespiratory fitness in children [26, 27].

### Statistical analysis

A complete case analysis was applied. Forty out of 882 participants at baseline moved or changed schools within the 6-month anthelmintic treatment follow-up, and hence, did not participate in the latter cross-sectional survey. Statistical analyses were performed with STATA version 14.1 (StataCorp; College Station, TX, USA). Statistical significance was defined as a two-sided p-value<0.05.

Descriptive statistics include counts, percentages for categorical variables and, means, and standard deviations (SD) for continuous variables. The categorization of DM status by sex is described according to the American Diabetes Association cutoffs for HbA1c. The baseline prevalence of the different gastrointestinal tract infections is presented for the different schools separately. The characteristics of covariates at baseline are presented stratified for infected and non-infected children. To assess the independent association between gastrointestinal tract infections and HbA1c measurement (treated as continuous numerical data) at baseline, linear mixed regressions models with random intercepts for schools were computed. Models were *a priori* adjusted for factors previously shown to be associated with infections and glycemia or diabetes, and therefore with a potential role as confounders: age, sex, SES, Hb, height, weight, BMI, systolic and diastolic blood pressure, physical activity, VO_2_ max, and body temperature. As a sensitivity analysis, we also omitted weight, BMI, physical activity, VO_2_ max, anemia and blood pressure from the models as they are potential mediators of infection effects on glycemia or correlates of glycemia. All models were run (i) by adding each infection separately without excluding children with other infections; (ii) by adding each infection separately and excluding children with other infections; (iii) by adding all infection variables simultaneously and; and (iv) by adding groups of infections. We also assessed dose-response effects on HbA1c for infectious agents, especially *A*. *lumbricoides*, where data on intensity of infection was available. To assess the independent effect of anthelmintic treatment on changes in HbA1c level between baseline and the 6-month anthelmintic treatment follow-up among children from schools without lifestyle intervention and who were infected at baseline, linear mixed regression models with random intercepts for schools were built. Models were *a priori* adjusted for age, sex, SES, Hb, height, weight, BMI, diastolic and systolic blood pressure, physical activity, VO_2_ max, and body temperature, considering information from both time points, as appropriate. Longitudinal models were re-run among subjects infected at baseline but not at follow-up, to differentiate between the effect of the anthelmintic treatment itself and the effect of resolved infection on change in HbA1c. Models were also run for children infected with nematodes and for children with any gastrointestinal tract infection separately.

## Results

**Fig 1:**
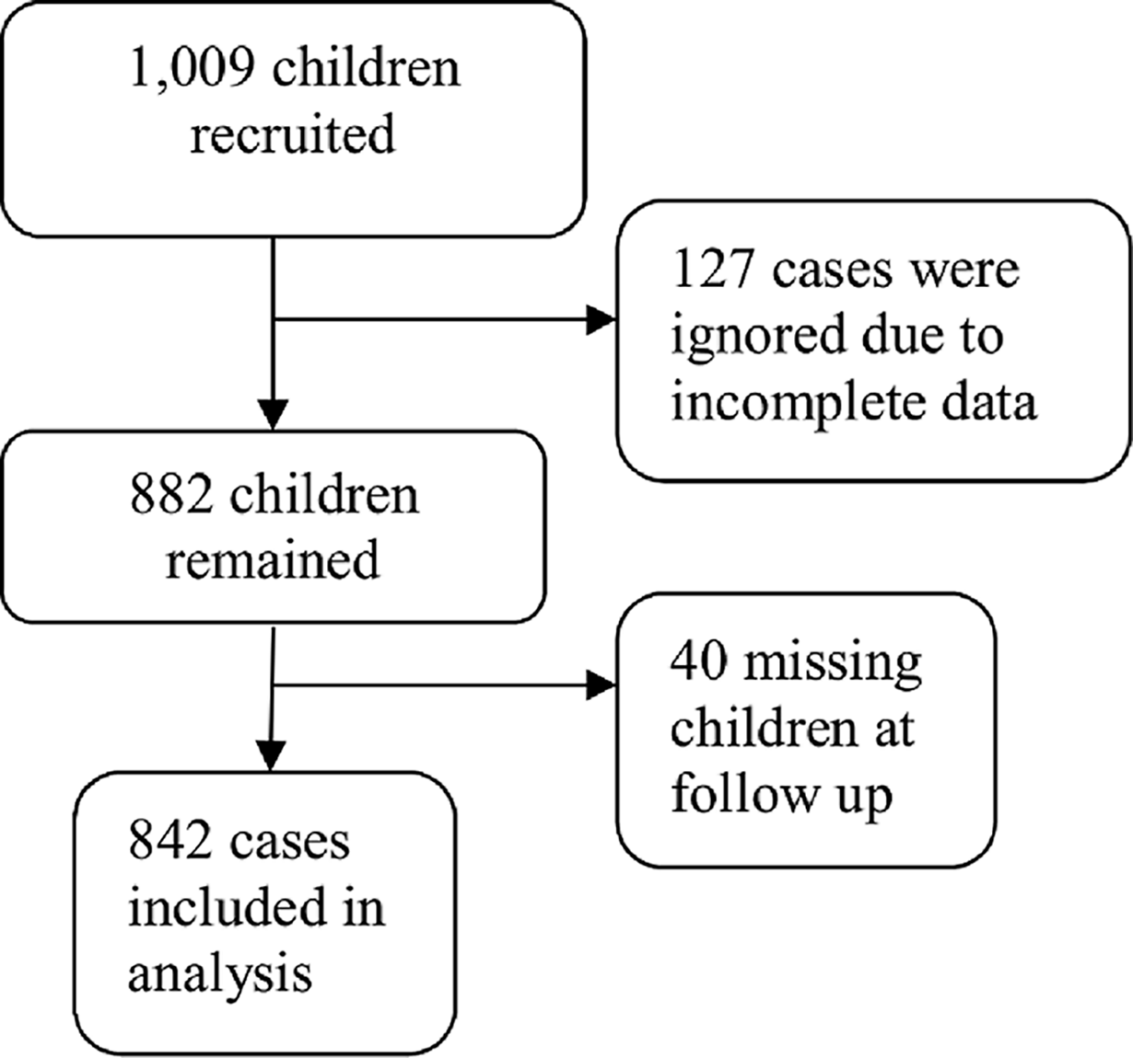
Children retained in the study sample for complete case analysis.

**Fig 2:**
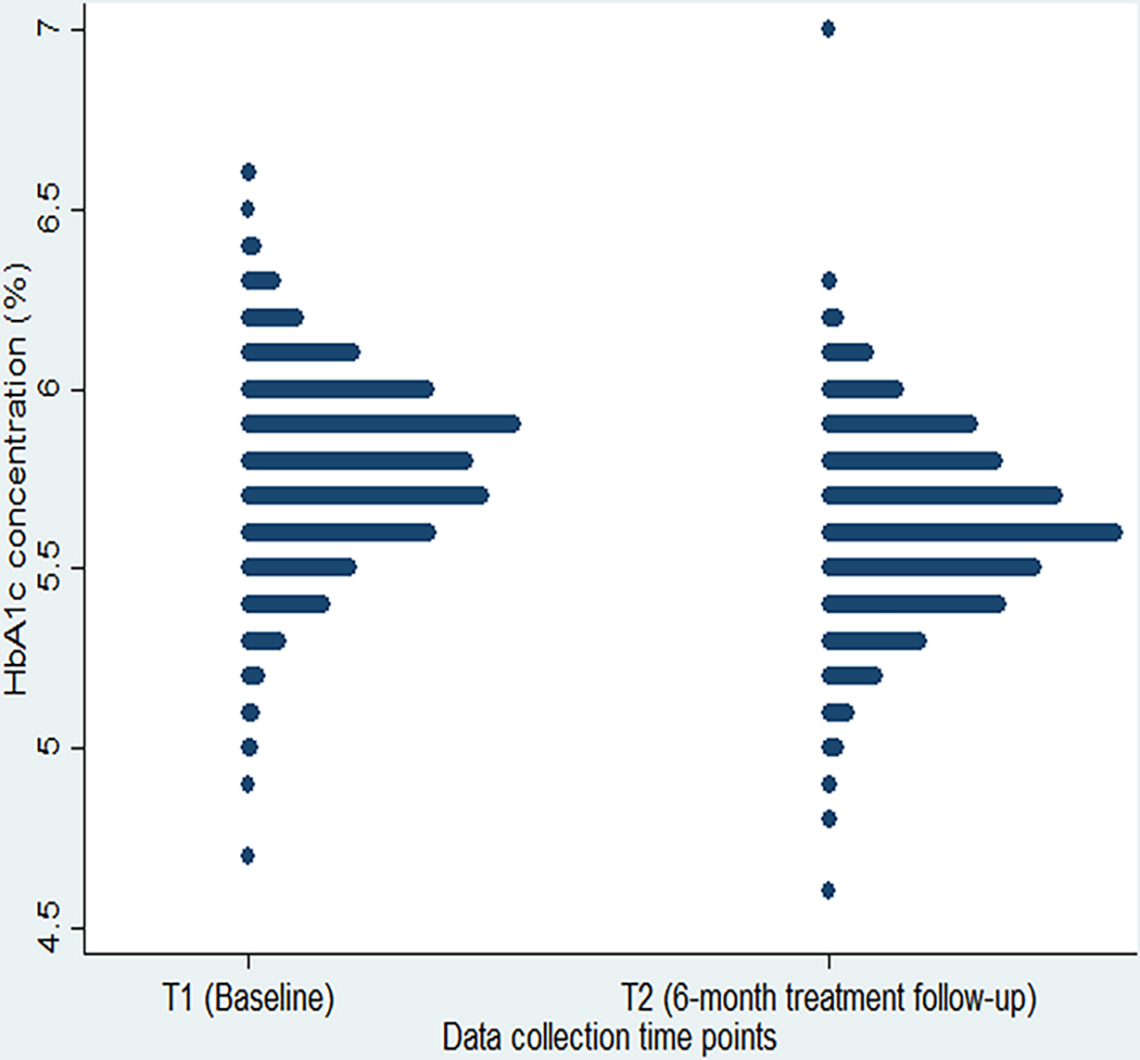
Distribution of HbA1c measured at baseline and 6 month follow-up in the total study sample, irrespective of the intervention obtained(N = 842).

Complete data records including the baseline and 6-month anthelmintic treatment follow-up surveys were available from 842 children (Fig 1). Fig 2 shows the distribution of HbA1c at baseline and at the 6-month follow-up for the total study sample of 842 children irrespective of the intervention that they obtained. There was a small shift towards lower HbA1c levels at follow-up (p<0.001), reflecting the lifestyle intervention in some schools. The results of quality control tests underline the validity of the HbA1c data. First, HbA1c results did not depend on the day of examination (p = 0.222), body temperature (p = 0.327), or ambient temperature (p = 0.217) (S1 Fig). Second, results from the weekly calibration with identical control 1 and control 2 are presented in S2 Table. At baseline, the overall mean HbA1c level of participants was 5.79% with SD of 0.25. The prevalence of prediabetes and diabetes according to baseline is presented in S3 Table. A high prevalence of preDM was observed with 605 (71.8%) of children having preDM HbA1c levels. Three children (0.4%) exhibited HbA1c results ≥6.5% at baseline and were offered diagnostic follow-up for DM. The characteristics of the study population and its univariate association with HbA1c are presented in S4 Table.

Table 1 shows the prevalence of gastrointestinal tract infections in the study schools at baseline. *H*. *pylori* was the predominant infection (416 children with a positive RDT result, 49.4%). At the unit of the school, the prevalence of *H*. *pylori* ranged from 27% to 62%.

**Table 1:** Baseline prevalence (%) of gastrointestinal tract infections, stratified by schools.

Schools and infection status	School 1(N = 89)	School 2(N = 168)	School 3^1^(N = 81)	School 4^1^(N = 102)	School 5(N = 82)	School 6(N = 84)	School 7^1^(N = 143)	School 8^1^(N = 93)	Total N,%(842,100.0)
**Nematodes(n,%)**	**1,1.1**	**133,79.2**	**67,82.7**	**4,3.9**	**1,1.2**	**4,4.8**	**42,29.4**	**9,9.7**	**261,31.0**
*Ascaris lumbricoides*(%)	1.1	62.5	74.1	1.0	0	3.6	25.9	4.3	**211,25.1**
*Trichuris trichiura*(%)	0	66.7	67.9	0	1.2	0	2.1	2.2	**137,16.3**
*Enterobius vermicularis*(%)	0	1.2	3.7	2.0	1.2	2.4	2.8	4.3	**18,2.1**
**Trematodes(n,%)**	**8,9.0**	**11,6.6**	**1,1.2**	**1,1.0**	**6,7.3**	**5,6.0**	**9,6.3**	**5,5.4**	**46,5.5**
*Schistosoma mansoni*(%)	0	3.0	0	1.0	1.2	0	0.7	0	**8,1.0**
*Schistosoma haematobium*(%)*	9.0	3.6	1.2	0	6.1	6.0	5.6	5.4	**38,4.5**
**Intestinal protozoa(n,%)**	**13,14.6**	**30,17.9**	**11,13.6**	**7,6.9**	**11,13.4**	**9,10.7**	**24,16.8**	**9,9.7**	**114,13.5**
*Cryptosporidium parvum*(%)	3.4	1.8	1.2	1.0	4.9	4.8	4.2	2.2	**21,2.4**
*Giardia intestinalis*(%)	11.2	16.1	12.4	5.9	9.8	9.5	14.0	7.5	**96,11.4**
***Helicobacter pylori***(%)	**62,69.7**	**111,66.1**	**50,61.7**	**53,52.0**	**35,42.7**	**23,27.4**	**42,29.4**	**40,43.0**	**416,49.4**

^1^ Schools without intervention related to health education, nutrition, and physical activity

N Number of children in each school

n Number of infected children in each school and parasite group

*S. *haematobium* infections only detected with hemastix strips

The second most common infections were the soil-transmitted helminths *A*. *lumbricoides* and *T*. *trichiura*. Two out of eight schools showed very high prevalence of *A*. *lumbricoides* infection (62.5% and 74.1%), there was a moderate infection prevalence in a third school (25.9%), while the prevalence in the five remaining schools were below 5%. High prevalence of *T*. *trichiura* infection was observed in the same two schools where the prevalence of *A*. *lumbricoides* prevalence was high (66.7% and 67.9%, respectively), while the prevalence of *T*. *trichiura* was below 3% in the remaining six schools.

In all schools, infection rates were low to very low or even undetectable for intestinal protozoa (*Cryptosporidium* spp. 1–5%; *G*. *intestinalis* 6–17%), the nematode *E*. *vermicularis* (1–5%), and the trematodes *S*. *mansoni* (1–3%; detected by POC-CCA urine cassette test) and *S*. *haematobium* (0%).

Table 2 simply compares the characteristics of participants with and without a specific gastrointestinal tract infection. Except for *H*. *pylori*, the proportion of children with low SES was higher among infected children compared to their non-infected counterparts. Infections with nematodes and *G*. *intestinalis* were more common in males, whereas *C*. *parvum* infection was more common in females. Infected children were, on average, older than their non-infected peers. Nevertheless, children with an *A*. *lumbricoides*, *T*. *trichiura*, and *E*. *vermicularis* infection had lower height, weight, and BMI compared to non-infected children. However, children infected with *A*. *lumbricoides*, *T*. *trichiura*, and *H*. *pylori* reported higher physical activity, but did not differ with regard to cardiorespiratory fitness. Concerning anemia and HbA1c, no clear pattern of association was evident from the univariate analysis.

**Table 2:** Distribution of characteristics of participants at baseline, by presence or absence of specific gastrointestinal tract infections.

Covariates	*A*.* lumbricoides*		*T*.* trichiura*		*E*.* vermicularis*		*S*.* mansoni*		*S*.* haematobium*		*C*.* parvum*		*G*.* intestinalis*		*H*.* pylori*	
	No	Yes	No	Yes	No	Yes	No	Yes	No	Yes	No	Yes	No	Yes	No	Yes
Low SES: N, %	189,30.0	147,70.0	218,32.6	118,68.2	326,39.6	10,55.6	332,39.8	4,50.0	316,39.3	20,52.6	329,40.0	7,33.3	291,39.0	45,46.9	172,40.4	164,39.4
Female: N, %	322,51.4	94,45.5	342,51.4	74,42.7	409,50.1	7,38.9	412,49.9	4,50.0	392,48.8	24,63.1	401,48.8	15,71.4	374,50.1	42,43.8	205,48.4	211,51.4
Age,years:N, mean,SD	631,10.7,0.972	211,11.2,0.865	669,10.7,0.987	173,11.2,0.789	824,10.8,0.969	18,10.9,1.017	834,10.8,0.970	8,11.6,0.721	804,10.8,0.953	38,11.2,1.239	821,10.8,0.972	21,10.8,0.905	746,10.8,0.964	96,11.1,0.994	426,10.8,1.012	416,10.9,0.926
Height, cm: N, mean, SD	631,133.8,7.083	211,131.5,6.830	669,133.8,6.933	173,130.8,7.164	824,133.3,7.063	18,130.0,7.547	834,133.2,7.064	8,139.4,6.931	804,133.1,7.034	38,134.7,8.045	821,133.1,7.039	21,134.8,8.756	746,133.2,7.067	96,133.3,7.257	426,133.7,7.214	416,132.8,6.929
Weight, kg: N, mean, SD	631,31.3,8.048	211,28.1,5.580	669,31.3,7.901	173,27.4,5.463	824,30.6,7.660	18,26.9,4.662	834,30.4,7.634	8,34.1,5.944	804,30.5,7.668	38,30.9,6.747	821,30.4,7.596	21,32.7,8.615	746,30.5,7.628	96,30.5,7.650	426,30.0,7.938	416,30.9,7.276
BMI, kg/m^2^: N, mean, SD	631,17.3,3.254	211,16.1,2.102	669,17.3,3.213	173,15.9,1.930	824,17.0,3.066	18,15.8,1.626	834,17.0,3.048	8,17.6,3.108	804,17.0,3.084	38,16.9,2.168	821,17.0,3.057	21,17.7,2.602	746,17.0,3.054	96,17.0,3.010	426,3.2,3.186	416,2.9,2.898
VO_2_ max,ml/kg/min: N,mean, SD	631,45.9,5.080	211,45.5,5.098	669,45.8,5.099	173,45.8,5.055	824,45.8,5.048	18,46.6,6.729	834,45.8,5.073	8,46.4,6.805	804,45.9,5.081	38,44.3,5.210	821,45.9,5.081	21,43.8,5.050	746,46.1,5.027	96,45.5,5.562	426,46.0,5.375	416,45.9,4.781
Low physical activity: N,%	250,39.6	56,26.5	271,40.5	35,20.2	297,36.0	9,50.0	302,36.2	4,50.0	292,36.3	14,36.8	298,36.3	8, 38.1	274,36.7	32,33.3	176,41.3	130,31.3
Hb, g/l,:N, mean, SD	631,12.3,0.948	211,11.9,0.916	669,12.3,0.963	173,11.9,0.834	824,12.2,0.948	18,12.1,0.108	834,12.2,0.949	8,12.3,0.112	804,12.2,0.947	38,12.5,0.989	821,12.2,0.946	21,12.3,0.112	746,12.2,0.964	96,12.3,0.842	426,12.3,0.959	416,12.2,0.939
HbA1c %: N, mean, SD	631,5.8,0.245	211,5.7,0.255	669,5.8,0.243	173,5.7,0.270	824,5.8,0.249	18,5.7,0.266	834,5.8,0.250	8,5.9,0.225	804,5.8,0.250	38,5.8,0.229	821,5.8,0.249	21,5.8,0.264	746,5.8,0.245	96,5.8,0.284	426,5.8,0.246	416,5.8,0.253

N Number of children

The results from the multivariable linear regression models of the cross-sectional association of single or grouped infections with HbA1c are presented in Table 3. We observed a positive association between *H*. *pylori* infection and HbA1c, irrespective of adjustments for other infections (β = 0.040; 95% confidence interval (CI) 0.006–0.074). No significant association of HbA1c with any other infectious agent or infection group was observed. Omitting covariates from the multivariable regression models that are potential mediators of infection effects on glycemia (physical activity, physical fitness, weight, BMI, and anemia) or correlated outcomes (blood pressure) did not materially alter the results presented for the fully adjusted models (S5 Table). Excluding children with DM at baseline or at the 6-month anthelmintic treatment follow-up did not materially alter the findings (S6 Table). In addition, we were not able to show a statistically significant dose-response relationship between intensity of *A*. *lumbricoides* and *T*. *trichiura* infection and HbA1c levels, albeit adjusted HbA1c levels were highest in children with most intense infections (S7 Table).

**Table 3:** Adjusted associations of infection with HbA1c at baseline.

**Single infections and infection groups**	**All with respective infection**^**1**^			**Only respective infection**^**2**^			**Mutually adjusted for other infections or groups**^**3**^		
	**N**	β*	**95% CI**	**N**	β*	**95% CI**	**N**	β*	**95% CI**
**Nematodes**	842	-0.018	-0.069–0.032	343	-0.039	-0.115–0.037	842	-0.027	-0.079–0.024
*A*.* lumbricoides*	842	-0.021	-0.070–0.029	307	-0.039	-0.133–0.055	842	-0.029	-0.080–0.023
*T*.* trichiura*	842	0.000	-0.060–0.060	280	0.061	-0.208–0.330	842	0.002	-0.061–0.066
*E*.* vermicularis*	842	-0.053	-0.164–0.058	284	-0.079	-0.251–0.093	842	-0.057	-0.168–0.054
**Trematodes**	842	0.013	-0.058–0.084	296	0.012	-0.097–0.120	842	0.012	-0.059–0.083
*S*.* mansoni*	842	0.033	-0.132–0.199	278	0.069	-0.387–0.525	842	0.029	-0.137–0.195
*S*.* haematobium*	842	0.008	-0.070–0.086	295	0.011	-0.102–0.123	842	0.006	-0.072–0.084
**Protozoa**	842	-0.006	-0.052–0.041	283	-0.126	-0.314–0.062	842	-0.005	-0.052–0.042
*C*.* parvum*	842	-0.020	-0.122–0.083	283	-0.121	-0.310–0.067	842	-0.030	-0.133–0.074
*G*.* intestinalis*	842	0.005	-0.045–0.055	305	0.001	-0.093–0.095	842	0.006	-0.045–0.057
***H*.* pylori***	**842**	**0.040**	**0.006–0.074**	488	0.041	-0.003–0.085	**842**	**0.041**	**0.007–0.075**
**Nematode infections**	**All with respective infection**^**1**^								
	**N**	β*	**95% CI**						
Only nematodes	842	-0.057	-0.124–0.011						
Nematodes and other infections	842	0.013	-0.046–0.070						
Only other infections	842	0.016	-0.024–0.055						

* Beta coefficients reflect the adjusted mean difference HbA1c (%) between children with and without the respective infection. Differences that are statistically significantly different (p<0.05) are marked in bold.

^1^Single and group infection models as well as nematode infection models are adjusted for school age, sex, socioeconomic status (SES), hemoglobin (Hb) level, height, weight, BMI, systolic and diastolic blood pressure, physical activity, physical fitness, body temperature on the day of the HbA1c test

^2^Children with other infections are excluded from this analysis

^3^Mutually adjusted models include either all single infections or all infection groups; *H*. *pylori* is included in single infection and infection group models

Results pertaining to the association between albendazole treatment and change in HbA1c level at the 6-month treatment follow-up are presented in Table 4. The analysis is restricted to children from schools not subjected to lifestyle interventions given the observed slight decrease in HbA1c in the total study sample. Furthermore, only children with any infection or with nematode infection at baseline, respectively, were included. The regression analyses point to statistically non-significant increases in HbA1c concentrations at the 6-month treatment follow-up. The findings from multivariable regression model excluding covariates that could be potential mediators of infection effects on glycemia or correlated outcomes (weight, BMI, anemia, physical activity, physical fitness, and blood pressure) point to generally weaker and still statistically non-significant results (S8 Table).

**Table 4:** Adjusted^1^ estimate of average change in HbA1c (follow-up minus baseline) among children infected at baseline and visiting schools without lifestyle intervention.

Infections exposures	N	Estimate average change in HbA1c (%)	95% CI
**Nematode infections**			
All subjects with a nematode infection at baseline, adjusted for the presence of infection of any type at baseline and follow-up	414	0.049	-0.018–0.117
Subjects with a nematode infection at baseline, but without any infection at follow-up	217	0.025	-0.008–0.108
**Any infection**			
All subjects with any infection at baseline, adjusted for the presence of infection at follow-up	260	0.070	-0.008–0.148
Subjects with any infection at baseline, but without any infection at follow-up	103	0.054	-0.055–0.164

^1^All models were adjusted for schools, age, sex, socioeconomic status (SES), hemoglobin (Hb) level, weight, height BMI, physical activity, VO_2_ max, and body temperature, systolic and diastolic blood pressure at baseline and follow-up

## Discussion

To our knowledge this is the first investigation examining the cross-sectional association of a broad spectrum of gastrointestinal tract infections with glycemia in school-aged children and assessing the impact of anthelmintic treatment on the change in HbA1c values. We observed a positive association between *H*. *pylori* infection and HbA1c, while no statistically significant relationship was observed with any other type of infection.

Some animal experiments [28] and human epidemiologic studies [10, 11, 13, 15] have shown helminth infections to lower the blood sugar level and inhibit the development of type 1 DM as well as type 2 DM. An inverse relationship between lymphatic filariasis and both type 1 and type 2 DM was reported from India [10, 11]. Having a previous schistosome infection exhibited a strong protective effect against DM in the People’s Republic of China [12]. *Strongyloides stercoralis* infection seemed to be associated with a reduced risk of type 2 DM in adult Australians [13]. Soil-transmitted helminth infections were linked with an improvement of insulin sensitivity in Indonesia [15]. Diabetic patients in Turkey were found to have a lower prevalence of parasitic disease than their healthy counterparts [16]. In contrast, a positive association was found between *S*. *stercoralis* infection and DM in Brazil, where it was also found that such infections were associated with a high mortality risk among poorly controlled DM patients [14]. A study conducted by Hakim and colleagues reported a high rate of *G*. *intestinalis* infection among DM patients [29]. For trematode infections, positive association with HbA1c concentrations were reported from several studies [12, 30, 31]. The cross-sectional nature of these studies precludes casual inference.

*H*. *pylori* is one of the most common human pathogens causing gastrointestinal inflammation. Potential underlying mechanisms linking *H*. *pylori* infection and HbA1c levels and DM may include a disturbance of glucose and lipid absorption by the inflamed gastrointestinal tissue. *H*. *pylori* infections may also alter host metabolic homeostasis by affecting appetite regulation and energy expenditure through altered balance of ghrelin and leptin secretion, leading to over-eating and metabolic syndrome pathogenesis. The mediating role of gut microbiota alterations remains unknown [32]. The reported associations between *H*. *pylori* infection and DM remain inconsistent. The positive association reported among school children in the present study corroborates findings from two large cross-sectional national surveys conducted by Chen and Blaser in American population samples (one aged ≥18 years and one aged ≥3 years) and a Taiwanese study in adults, which all found that *H*. *pylori* infections were associated with higher mean HbA1c levels [33, 34]. Several smaller outpatient clinic or hospital based studies in Turkey, Pakistan, and Qatar among adults aged 18 years and above showed a higher prevalence of *H*. *pylori* infection in diabetic patients than non-DM control groups [35–37]. Other studies failed to find a positive association between *H*. *pylori* and HbA1c or DM [38–40].

In fact, DM patients were found to have higher rates of *H*. *pylori* eradication therapy according to national health insurance data from Taiwan. *H*. *pylori* eradication treatment success was found to be lower in DM compared to non-DM patients [41, 42]. Future intervention studies for the treatment of *H*. *pylori* should systematically consider changes in glycemia to shed light on the potential etiologic role of *H*. *pylori* in DM development. Some studies indicated an improvement of mean HbA1c and insulin resistance in patients with type 2 DM after *H*. *pylori* treatment [43, 44].

We did not observe a statistically significant increase in HbA1c after anthelmintic treatment with albendazole in children harboring nematode infections at baseline, possibly as a result of sample size limitations. The observed direction of the effect is in line with the reported shift towards a Th2 response in helminth-infected individuals. A number of clinical trials with helminth or helminth antigen therapy have reported promising results in inflammatory bowel diseases [45, 46], multiple sclerosis [47], rheumatoid arthritis [48]. After deworming, which triggers several hyper-inflammatory processes and shifts immune responses from Th2 to Th1, groups of children treated with either albendazole or mebendazole (against soil-transmitted helminthiasis) or praziquantel (against schistosomiasis) had a higher positive response to the skin-prick test and allergy related symptoms [49, 50]. Nevertheless, other studies emphasized that anthelmintic treatment did not have an effect on clinical eczema and asthmatic severity scores [51, 52].

Given that in our study the highest increase in HbA1c after albendazole treatment was observed in children with non-nematode parasite infection, additional research is needed to understand the effect of the anthelmintic drugs on human glucose metabolism. Yet, our results are aligned with the first publication from a randomized placebo-controlled trial in Indonesia, which showed no effect of albendazole treatment on insulin resistance [53].

Our study has several strengths. First, the study population exhibited sufficient prevalence range for at least some of the infectious agents investigated to allow for efficient interrogation of the study objective. Second, the detailed characterization of children allowed us to assess independent associations of parasite infections with glycemia and limiting residual confounding. To analyze the SES of study participants, we chose multiple correspondence analysis (MCA) based on household characteristics and assets ownership over more traditional methods thereby minimizing measurement error related to the different calculation methods of income and consumption, recall bias, and seasonal variation of income and expenditure. Third, we used internationally certified HbA1c testing (Alere Technologies), regularly calibrated with standard control procedure. Ehehalt et al. showed that the measurement of HbA1c was a reliable criterion for children and adolescents to diagnose the onset of childhood type 1 DM [54]. In addition, the POC HbA1c test is an accepted screening instrument for pre-DM and type 2 DM [55, 56]. We carefully evaluated potential measurement error in HbA1c in the light of the observed high prevalence of pre-DM. We demonstrated the absence of correlations with external temperature, body temperature, and examination date. In addition, all models were adjusted for the concentration of Hb, a potentially important confounder, which was assessed with the widely used HemoCue Hb 301 system.

We also acknowledge some limitations of our study. Reverse causation remains a problem related to the cross-sectional nature of our main analysis. The low prevalence for some infections limited statistical power for the analyses. The association between *H*. *pylori* infection and HbA1c is no longer statistically significant if the p-values are adjusted for the number of infections investigated (n = 8). Additionally, the co-infections [3] may in part mask opposite effects of different parasites on HbA1c. Examining only one stool sample has a low diagnostic accuracy due to the day-to-day and intra-specimen variation in helminth egg output. To partially remedy this shortcoming, test specificity was increased by preparing duplicate Kato-Katz thick smears from each stool sample. We observed a very high rate of preDM in the children studied, which may limit the generalizability of the observed associations. Despite the fact that the Alere HbA1c testing is minimally affected by hemoglobinopathies, we cannot assess any influence in the absence of genotyping results. Selection bias related to the complete case analysis approach cannot be excluded but the very high participation rate at baseline and the 6-month anthelmintic treatment follow-up (5% drop-out rate), and the relatively low rate of children not providing stools (15%) are unlikely to have substantially altered the results.

In conclusion, the positive cross-sectional association of *H*. *pylori* infections with glycemia is consistent with a potential role of this highly prevalent bacterium in DM in LMICs. The direction and causality of the association warrants further scientific inquiry in the context of longitudinal studies and biobanks that focus on specific parasites and integrate immunity as well as other biomarkers to improve mechanistic understanding of parasite-glycemia associations and the potential impact of deworming programs on DM prevalence.

## Supporting information

S1 STROBE checklist Association between gastrointestinal tract infections and glycated hemoglobin in school children of poor neighborhoods in Port Elizabeth, South Africa. (DOC)

S1 Fig Plot of individual baseline HbA1c results versus date of examination, body temperature and ambient temperature in all schools. (PDF)

S1 Table Multiple Component Analysis (MCA) of SES of the participants. (PDF)

S2 Table HbA1c measurements in control probes integrated into baseline and 6-month follow up assessment. (PDF)

S3 Table Distribution of prediabetes and diabetes based on HbA1c cutoff^1^ at baseline, by gender. (PDF)

S4 Table Demographic profile of participants and HbA1c assessment. (PDF)

S5 Table Adjusted association of helminth infections and HbA1c measurement at baseline, omitting adjustment for potential mediators and correlated outcomes. (PDF)

S6 Table Adjusted association of helminth infections and HbA1c measurement at baseline—DM cases excluded. (PDF)

S7 Table Adjusted association of *A*. *lumbricoides* and *T*. *trichiura* infection intensities and HbA1c measurement at baseline. (PDF)

S8 Table Adjusted estimate of average change in HbA1c (follow-up minus baseline) among children infected at baseline and visiting schools without lifestyle intervention, omitting adjustment for potential mediators and correlated outcomes. (DOCX)

## References

[1] Hotez PJ, Alvarado M, Basáñez M-G, Bolliger I, Bourne R, Boussinesq M, et al The Global Burden of Disease Study 2010: Interpretation and Implications for the Neglected Tropical Diseases. PLoS Negl Trop Dis. 2014;8(7):e2865 doi: 10.1371/journal.pntd.0002865 25058013 10.1371/journal.pntd.0002865 PMC4109880

[2] Bardgett RD, van der Putten WH. Belowground biodiversity and ecosystem functioning. Nature. 2014;515(7528):505–11. doi: 10.1038/nature13855 25428498 10.1038/nature13855

[3] Müller I, Yap P, Steinmann P, Damons BP, Schindler C, Seelig H, et al Intestinal parasites, growth and physical fitness of schoolchildren in poor neighbourhoods of Port Elizabeth, South Africa: a cross-sectional survey. Parasites & Vectors. 2016;9(1):488 doi: 10.1186/s13071-016-1761-5 27595566 10.1186/s13071-016-1761-5 PMC5011914

[4] Freeman MC, Ogden S, Jacobson J, Abbott D, Addiss DG, Amnie AG, et al Integration of water, sanitation, and hygiene for the prevention and control of neglected tropical diseases: a rationale for inter-sectoral collaboration. PLoS Negl Trop Dis. 2013;7(9):e2439 Epub 2013/10/03. doi: 10.1371/journal.pntd.0002439 ; PubMed Central PMCID: PMCPMC3784463. 24086781 10.1371/journal.pntd.0002439 PMC3784463

[5] Kassebaum N, Arora M, Barber R, Bhutta Z, Brown J, Carter A, et al Global, regional, and national disability-adjusted life-years (DALYs) for 315 diseases and injuries and healthy life expectancy (HALE), 1990–2015: a systematic analysis for the Global Burden of Disease Study 2015. Lancet. 2016;388(10053):1603–58. Epub 2016/10/14. doi: 10.1016/S0140-6736(16)31460-X ; PubMed Central PMCID: PMCPMC5388857. 27733283 10.1016/S0140-6736(16)31460-X PMC5388857

[6] IDF. Diabetes Atlas: International Diabetes Federation; 2014 [updated 2014/12/04/19:17:10; cited 2014 October 4]. Available from: http://www.idf.org/diabetesatlas.

[7] Lindblom R, Ververis K, Tortorella SM, Karagiannis TC. The early life origin theory in the development of cardiovascular disease and type 2 diabetes. Molecular biology reports. 2015;42(4):791–7. Epub 2014/10/02. doi: 10.1007/s11033-014-3766-5 . 25270249 10.1007/s11033-014-3766-5

[8] Lamain-de Ruiter M, Kwee A, Naaktgeboren CA, de Groot I, Evers IM, Groenendaal F, et al External validation of prognostic models to predict risk of gestational diabetes mellitus in one Dutch cohort: prospective multicentre cohort study. Bmj. 2016;354:i4338 Epub 2016/09/01. doi: 10.1136/bmj.i4338 . 27576867 10.1136/bmj.i4338

[9] Bhattacharjee S, Kalbfuss N, Prazeres da Costa C. Parasites, microbiota and metabolic disease. Parasite immunology. 2016 Epub 2016/10/08. doi: 10.1111/pim.12390 . 27716947 10.1111/pim.12390

[10] Aravindhan V, Mohan V, Surendar J, Muralidhara Rao M, Pavankumar N, Deepa M, et al Decreased Prevalence of Lymphatic Filariasis among Diabetic Subjects Associated with a Diminished Pro-Inflammatory Cytokine Response (CURES 83). PLoS Negl Trop Dis. 2010;4(6). doi: 10.1371/journal.pntd.0000707 20559443 10.1371/journal.pntd.0000707 PMC2886036

[11] Aravindhan V, Mohan V, Surendar J, Rao MM, Ranjani H, Kumaraswami V, et al Decreased Prevalence of Lymphatic Filariasis Among Subjects with Type-1 Diabetes. Am J Trop Med Hyg. 2010;83(6):1336–9. doi: 10.4269/ajtmh.2010.10-0410 21118945 10.4269/ajtmh.2010.10-0410 PMC2990055

[12] Chen Y, Lu J, Huang Y, Wang T, Xu Y, Xu M, et al Association of Previous Schistosome Infection With Diabetes and Metabolic Syndrome: A Cross-Sectional Study in Rural China. The Journal of Clinical Endocrinology & Metabolism. 2012;98(2):E283–E7. doi: 10.1210/jc.2012-2517 23275524 10.1210/jc.2012-2517

[13] Hays R, Esterman A, Giacomin P, Loukas A, McDermott R. Does Strongyloides stercoralis infection protect against type 2 diabetes in humans? Evidence from Australian Aboriginal adults. Diabetes research and clinical practice. 2015;107(3):355–61. Epub 2015/02/07. doi: 10.1016/j.diabres.2015.01.012 . 25656764 10.1016/j.diabres.2015.01.012

[14] Mendonça SCL, Gonçalves-Pires MdRF, Rodrigues RM, Ferreira Á Jr, Costa-Cruz JM. Is there an association between positive Strongyloides stercoralis serology and diabetes mellitus? Acta Tropica. 2006;99(1):102–5. doi: 10.1016/j.actatropica.2006.06.006 16872576 10.1016/j.actatropica.2006.06.006

[15] Wiria AE, Hamid F, Wammes LJ, Prasetyani MA, Dekkers OM, May L, et al Infection with Soil-Transmitted Helminths Is Associated with Increased Insulin Sensitivity. PloS one. 2015;10(6):e0127746 doi: 10.1371/journal.pone.0127746 PubMed PMID: PMC4464734. 26061042 10.1371/journal.pone.0127746 PMC4464734

[16] Nazligul Y, Sabuncu T, Ozbilge H. Is there a predisposition to intestinal parasitosis in diabetic patients? Diabetes Care. 2001;24(8):1503–4. Epub 2001/07/27. . 11473099 10.2337/diacare.24.8.1503-a

[17] Yap P, Müller I, Walter C, Seelig H, Gerber M, Steinmann P, et al Disease, activity and schoolchildren’s health (DASH) in Port Elizabeth, South Africa: a study protocol. BMC Public Health. 2015;15(1):1285 doi: 10.1186/s12889-015-2636-y 26700478 10.1186/s12889-015-2636-y PMC4690231

[18] World Health Organization. Preventive chemotherapy in human helminthiasis Geneva, Switzerland: 2006.

[19] Katz N, Chaves A, Pellegrino J. A simple device for quantitative stool thick-smear technique in Schistosomiasis mansoni. Revista do Instituto de Medicina Tropical de Sao Paulo. 1972;14(6):397–400. Epub 1972/11/01. . 4675644

[20] Knopp S, Mgeni AF, Khamis IS, Steinmann P, Stothard JR, Rollinson D. Diagnosis of soil-transmitted helminths in the era of preventive chemotherapy: effect of multiple stool sampling and use of different diagnostic techniques. PLoS Negl Trop Dis. 2008;2 doi: 10.1371/journal.pntd.0000331 18982057 10.1371/journal.pntd.0000331 PMC2570799

[21] Van den Bossche D, Cnops L, Verschueren J, Van Esbroeck M. Comparison of four rapid diagnostic tests, ELISA, microscopy and PCR for the detection of Giardia lamblia, Cryptosporidium spp. and Entamoeba histolytica in feces. Journal of microbiological methods. 2015;110:78–84. Epub 2015/01/24. doi: 10.1016/j.mimet.2015.01.016 . 25615719 10.1016/j.mimet.2015.01.016

[22] Choi J, Kim CH, Kim D, Chung SJ, Song JH, Kang JM, et al Prospective evaluation of a new stool antigen test for the detection of Helicobacter pylori, in comparison with histology, rapid urease test, (13)C-urea breath test, and serology. Journal of gastroenterology and hepatology. 2011;26(6):1053–9. Epub 2011/03/03. doi: 10.1111/j.1440-1746.2011.06705.x . 21362044 10.1111/j.1440-1746.2011.06705.x

[23] Colley DG, Binder S, Campbell C, King CH, Tchuem Tchuenté L-A, N'Goran EK, et al A Five-Country Evaluation of a Point-of-Care Circulating Cathodic Antigen Urine Assay for the Prevalence of Schistosoma mansoni. Am J Trop Med Hyg. 2013;88(3):426–32. doi: 10.4269/ajtmh.12-0639 PubMed PMID: PMC3592520. 23339198 10.4269/ajtmh.12-0639 PMC3592520

[24] Lorenzo C, Wagenknecht LE, Hanley AJ, Rewers MJ, Karter AJ, Haffner SM. A1C between 5.7 and 6.4% as a marker for identifying pre-diabetes, insulin sensitivity and secretion, and cardiovascular risk factors: the Insulin Resistance Atherosclerosis Study (IRAS). Diabetes Care. 2010;33(9):2104–9. Epub 2010/06/25. doi: 10.2337/dc10-0679 ; PubMed Central PMCID: PMCPmc2928372. 20573754 10.2337/dc10-0679 PMC2928372

[25] Filmer D, Pritchett LH. Estimating wealth effects without expenditure data—or tears: an application to educational enrollments in states of India. Demography. 2001;38(1):115–32. Epub 2001/03/03. . 11227840 10.1353/dem.2001.0003

[26] Leger L, Lambert J, Goulet A, Rowan C, Dinelle Y. [Aerobic capacity of 6 to 17-year-old Quebecois—20 meter shuttle run test with 1 minute stages]. Can J Appl Sport Sci. 1984;9(2):64–9. Epub 1984/06/01. . 6733834

[27] Léger LA, Lambert J. A maximal multistage 20-m shuttle run test to predict VO2 max. Eur J Appl Physiol Occup Physiol. 1982;49(1):1–12. 7201922 10.1007/BF00428958

[28] Hubner MP, Larson D, Torrero MN, Mueller E, Shi Y, Killoran K, et al Anti-Fc?R1 antibody injections activate basophils and mast cells and delay Type I diabetes onset in NOD mice. Clin Immunol. 2011;141(2):205–17. doi: 10.1016/j.clim.2011.08.004 21920822 10.1016/j.clim.2011.08.004 PMC3257875

[29] Hakim GD, Kızıltaş Ş, Çiftçi H, Göktaş Ş, Tuncer İ. The Prevalence of Giardia Intestinalis in Dyspeptic and Diabetic Patients. ISRN Gastroenterology. 2011;2011:580793 doi: 10.5402/2011/580793 PubMed PMID: PMC3168463. 21991517 10.5402/2011/580793 PMC3168463

[30] Geach T. Diabetes: Helminths improve insulin sensitivity and enhance M2 macrophage numbers in WAT of obese mice. Nature reviews Endocrinology. 2015;11(6):316 Epub 2015/04/29. doi: 10.1038/nrendo.2015.68 . 25917360 10.1038/nrendo.2015.68

[31] Soliman AT, El-Nawawy AA, El-Azzouni OF, Amer EA, Demian SR, El-Sayed MH. High prevalence of islet cell antibody and defective insulin release in children with schistosomiasis. Journal of Tropical Pediatrics. 1996;42(1):46–9. 8820621 10.1093/tropej/42.1.46

[32] Takeoka A, Tayama J, Yamasaki H, Kobayashi M, Ogawa S, Saigo T, et al Impact of Helicobacter pylori Immunoglobulin G Levels and Atrophic Gastritis Status on Risk of Metabolic Syndrome. PLOS ONE. 2016;11(11):e0166588 doi: 10.1371/journal.pone.0166588 27851820 10.1371/journal.pone.0166588 PMC5113018

[33] Chen Y, Blaser MJ. Association between gastric Helicobacter pylori colonization and glycated hemoglobin levels. J Infect Dis. 2012;205(8):1195–202. Epub 2012/03/20. doi: 10.1093/infdis/jis106 ; PubMed Central PMCID: PMCPMC3308905. 22427676 10.1093/infdis/jis106 PMC3308905

[34] Hsieh MC, Wang SS, Hsieh YT, Kuo FC, Soon MS, Wu DC. Helicobacter pylori infection associated with high HbA1c and type 2 diabetes. European journal of clinical investigation. 2013;43(9):949–56. Epub 2013/07/25. doi: 10.1111/eci.12124 . 23879740 10.1111/eci.12124

[35] Kayar Y, Pamukcu O, Eroglu H, Kalkan Erol K, Ilhan A, Kocaman O. Relationship between Helicobacter pylori Infections in Diabetic Patients and Inflammations, Metabolic Syndrome, and Complications. International journal of chronic diseases. 2015;2015:290128 Epub 2015/10/16. doi: 10.1155/2015/290128 ; PubMed Central PMCID: PMCPmc4590934. 26464868 10.1155/2015/290128 PMC4590934

[36] Devrajani BR, Shah SZ, Soomro AA, Devrajani T. Type 2 diabetes mellitus: A risk factor for Helicobacter pylori infection: A hospital based case-control study. International journal of diabetes in developing countries. 2010;30(1):22–6. Epub 2010/05/01. doi: 10.4103/0973-3930.60008 ; PubMed Central PMCID: PMCPmc2859280. 20431802 10.4103/0973-3930.60008 PMC2859280

[37] Bener A, Micallef R, Afifi M, Derbala M, Al-Mulla HM, Usmani MA. Association between type 2 diabetes mellitus and Helicobacter pylori infection. The Turkish journal of gastroenterology: the official journal of Turkish Society of Gastroenterology. 2007;18(4):225–9. Epub 2007/12/18. . 18080918

[38] Anastasios R, Goritsas C, Papamihail C, Trigidou R, Garzonis P, Ferti A. Helicobacter pylori infection in diabetic patients: prevalence and endoscopic findings. European journal of internal medicine. 2002;13(6):376 Epub 2002/09/13. . 12225782 10.1016/s0953-6205(02)00094-8

[39] Ko GT, Chan FK, Chan WB, Sung JJ, Tsoi CL, To KF, et al Helicobacter pylori infection in Chinese subjects with type 2 diabetes. Endocrine research. 2001;27(1–2):171–7. Epub 2001/06/29. . 11428708 10.1081/erc-100107178

[40] Stanciu OG, Trifan A, Sfarti C, Cojocariu C, Stanciu C. Helicobacter pylori infection in patients with diabetes mellitus. Revista medico-chirurgicala a Societatii de Medici si Naturalisti din Iasi. 2003;107(1):59–65. Epub 2004/02/06. . 14755971

[41] Tseng C-H. Diabetes, insulin use and Helicobacter pylori eradication: a retrospective cohort study. BMC gastroenterology. 2012;12(1):46 doi: 10.1186/1471-230x-12-46 22571603 10.1186/1471-230X-12-46 PMC3419616

[42] Sargyn M, Uygur-Bayramicli O, Sargyn H, Orbay E, Yavuzer D, Yayla A. Type 2 diabetes mellitus affects eradication rate of Helicobacter pylori. World journal of gastroenterology. 2003;9(5):1126–8. Epub 2003/04/30. doi: 10.3748/wjg.v9.i5.1126 ; PubMed Central PMCID: PMCPMC4611388. 12717872 10.3748/wjg.v9.i5.1126 PMC4611388

[43] Zojaji H, Ataei E, Sherafat SJ, Ghobakhlou M, Fatemi SR. The effect of the treatment of Helicobacter pylori infection on the glycemic control in type 2 diabetes mellitus. Gastroenterology and hepatology from bed to bench. 2013;6(1):36–40. Epub 2013/01/01. ; PubMed Central PMCID: PMCPMC4017496. 24834243 PMC4017496

[44] Gen R, Demir M, Ataseven H. Effect of Helicobacter pylori eradication on insulin resistance, serum lipids and low-grade inflammation. Southern medical journal. 2010;103(3):190–6. Epub 2010/02/06. doi: 10.1097/SMJ.0b013e3181cf373f . 20134372 10.1097/SMJ.0b013e3181cf373f

[45] Weinstock JV, Elliott DE. Translatability of helminth therapy in inflammatory bowel diseases. Int J Parasitol. 2013;43(3–4):245–51. Epub 2012/11/28. doi: 10.1016/j.ijpara.2012.10.016 ; PubMed Central PMCID: PMCPmc3683647. 23178819 10.1016/j.ijpara.2012.10.016 PMC3683647

[46] Summers RW, Elliott DE, Urban JF Jr., Thompson R, Weinstock JV. Trichuris suis therapy in Crohn's disease. Gut. 2005;54(1):87–90. Epub 2004/12/14. doi: 10.1136/gut.2004.041749 ; PubMed Central PMCID: PMCPMC1774382. 15591509 10.1136/gut.2004.041749 PMC1774382

[47] Fleming JO. Helminth therapy and multiple sclerosis. Int J Parasitol. 2013;43(3–4):259–74. Epub 2013/01/10. doi: 10.1016/j.ijpara.2012.10.025 . 23298637 10.1016/j.ijpara.2012.10.025

[48] Wammes LJ, Mpairwe H, Elliott AM, Yazdanbakhsh M. Helminth therapy or elimination: epidemiological, immunological, and clinical considerations. The Lancet Infectious diseases. 2014;14(11):1150–62. Epub 2014/07/02. doi: 10.1016/S1473-3099(14)70771-6 . 24981042 10.1016/S1473-3099(14)70771-6

[49] Flohr C, Tuyen LN, Quinnell RJ, Lewis S, Minh TT, Campbell J, et al Reduced helminth burden increases allergen skin sensitization but not clinical allergy: a randomized, double-blind, placebo-controlled trial in Vietnam. Clin Exp Allergy. 2010;40(1):131–42. Epub 2009/09/18. doi: 10.1111/j.1365-2222.2009.03346.x . 19758373 10.1111/j.1365-2222.2009.03346.x

[50] Wiria AE, Hamid F, Wammes LJ, Kaisar MMM, May L, Prasetyani MA, et al The Effect of Three-Monthly Albendazole Treatment on Malarial Parasitemia and Allergy: A Household-Based Cluster-Randomized, Double-Blind, Placebo-Controlled Trial. PLoS ONE. 2013;8(3):e57899 doi: 10.1371/journal.pone.0057899 PubMed PMID: PMC3602425. 23526959 10.1371/journal.pone.0057899 PMC3602425

[51] Ndibazza J, Muhangi L, Akishule D, Kiggundu M, Ameke C, Oweka J, et al Effects of deworming during pregnancy on maternal and perinatal outcomes in Entebbe, Uganda: a randomized controlled trial. Clin Infect Dis. 2010;50(4):531–40. Epub 2010/01/14. doi: 10.1086/649924 ; PubMed Central PMCID: PMCPMC2857962. 20067426 10.1086/649924 PMC2857962

[52] Almeida MCF, Lima GS, Cardoso LS, de Souza RP, Campos RA, Cruz AA, et al The Effect of Antihelminthic Treatment on Subjects with Asthma from an Endemic Area of Schistosomiasis: A Randomized, Double-Blinded, and Placebo-Controlled Trial. Journal of Parasitology Research. 2012;2012:296856 doi: 10.1155/2012/296856 PubMed PMID: PMC3425835. 22934153 10.1155/2012/296856 PMC3425835

[53] Tahapary DL, de Ruiter K, Martin I, Brienen EAT, van Lieshout L, Cobbaert CM, et al Effect of Anthelmintic Treatment on Insulin Resistance: A Cluster-Randomized Placebo-Controlled Trial in Indonesia. Clinical infectious diseases: an official publication of the Infectious Diseases Society of America. 2017 Epub 2017/05/05. doi: 10.1093/cid/cix416 . 28472383 10.1093/cid/cix416

[54] Ehehalt S, Gauger N, Blumenstock G, Feldhahn L, Scheffner T, Schweizer R, et al Hemoglobin A1c is a reliable criterion for diagnosing type 1 diabetes in childhood and adolescence. Pediatric diabetes. 2010;11(7):446–9. Epub 2010/02/13. doi: 10.1111/j.1399-5448.2009.00633.x . 20149124 10.1111/j.1399-5448.2009.00633.x

[55] Herman WH, Fajans SS. Hemoglobin A1c for the diagnosis of diabetes: practical considerations. Pol Arch Med Wewn. 2010;120(1–2):37–40. Epub 2010/02/13. . 20150843

[56] Rohlfing CL, Little RR, Wiedmeyer HM, England JD, Madsen R, Harris MI, et al Use of GHb (HbA1c) in screening for undiagnosed diabetes in the U.S. population. Diabetes Care. 2000;23(2):187–91. Epub 2000/06/27. . 10868829 10.2337/diacare.23.2.187

