# Supplementary material for: Association between gastrointestinal tract infections and glycated hemoglobin in school children of poor neighborhoods in Port Elizabeth, South Africa
Source: PLoS Negl Trop Dis. 2018 Mar 15;12(3):e0006332. doi: 10.1371/journal.pntd.0006332 (PMC5871004; doi:10.1371/journal.pntd.0006332)
Supplement: S1 STROBE checklist — (DOC) [file pntd.0006332.s001.doc]

STROBE Statement—checklist of items that should be included in reports of observational studies

|  | Item No | Recommendation |
| --- | --- | --- |
| **Title and abstract** | 1 | (*a*)A longitudinal study with two cross-sectional surveys |
|  |  | (*b*) We conducted a cross-sectional survey among 9- to 14-year-old school children in Port Elizabeth. Stool and urine samples were collected to assess infection status with parasitic worms, intestinal protozoa and bacterium. *Helicobacter pylori* was the predominant infectious agent and showed an independent positive association with HbA1c in a multivariate regression analysis (β=0.040, 95% confidence interval (CI) 0.006-0.073, p<0.05). No association between HbA1c and any other infectious agent was found, but we observed a statistically non-significant increase of mean HbA1c levels after albendazole administration. |
| Introduction | | |
| Background/rationale | 2 | A limited number of recently reviewed epidemiologic studies with inconsistent results investigated the cross-sectional association of different IDs, including lymphatic filariasis, schistosomiasis, strongyloidiasis, and soil-transmitted helminthiasis with DM or insulin sensitivity. In the present study, we followed up on these observations by studying the association of gastrointestinal tract infections due to helminths, intestinal protozoa, and the bacterium *Helicobacter pylori* with glycated hemoglobin (HbA1c) concentration in school children in the frame of the “Disease, Activity and Schoolchildren’s Health” (DASH) study in Port Elizabeth, South Africa. The study provided detailed information on physical activity, fitness, and socioeconomic status (SES) to consider as confounding factors on gastrointestinal tract infection status, and intensity of helminth infections to study a possible dose-response relationship; and on the longitudinal course of HbA1c upon selective anthelmintic treatment. |
| Objectives | 3 | The objective of this study was to investigate the association between common gastrointestinal tract infections and glycemia in school children from poor neighborhoods in South Africa. |
| Methods | | |
| Study design | 4 | The study was part of the 2-year longitudinal DASH study that consisted of three cross-sectional surveys. In each of the cross-sectional surveys, children’s gastrointestinal infections and other health parameters were assessed, including HbA1c, anthropometry, levels of physical fitness, cognitive performance, and psychosocial health. After each survey, helminth-infected children were treated with a single 400 mg oral dose of albendazole. |
| Setting | 5 | A total of 1,009 grade 4 children aged 9-14 years from eight non-fee paying primary schools were recruited in various parts of Port Elizabeth in the south-eastern part of South Africa. The baseline cross-sectional survey took place in March 2015. The current study considers data from this baseline survey and the anthelmintic treatment follow-up examination in September/October 2015. |
| Participants | 6 | (*a*) *Cross-sectional study*— Grade-4 primary school children of the selected schools were included in the study. Children with severe clinical signs and symptoms (e.g., severe fever, severe headache, dizziness, nausea, skin rashes, seizures, and diarrhea) or reported serious health problems, such as Crohn’s disease, liver or kidney diseases, or who participated in any other study were excluded. |
| Variables | 7 | Status with gastrointestional tract infections as predictors. The SES score, age, sex, BIM, height, weight, VO2max, physical activities score, body temperature, hemoglobin levels, as potential confounders. HbA1c measurements as outcomes. |
| Data sources/ measurement | 8* | ***Questionnaires and interviews.*** Standardized questionnaires available in both English and local languages (Afrikaans and Xhosa) were used to determine the SES of the children and their families. Volunteers fluent in relevant languages were trained to conduct these in-person interviews.  ***Clinical and anthropometric assessment.***Experienced nurses obtained a detailed medical history through physical examination of the whole body and evaluation of symptoms to assess current infections, anemia, jaundice, as well as signs and symptoms of protein energy malnutrition, general respiratory and gastrointestinal problems, allergies, and skin infections. Body temperature was measured using an infrared digital ear thermometer (TS7, Hi-Care International; Cape Town, South Africa). Blood pressure was measured once after the child had been seated for 5 min using validated oscillometric Omron® digital blood pressure monitor (Omron® M6 AC model; Hoofdoorp, Netherlands).  For the anthropometric measurements, shoes and sweaters were removed before standing on a digital weighting scale (Micro T7E electronic platform scale, Optima Electronics; George, South Africa). Body weight was measured once to the nearest 0.1 kg. Children’s height was assessed with a Seca stadiometer (Surgical SA; Johannesburg, South Africa), whereby the child was standing with the back erect, heals touching the wall, and shoulders relaxed. Body height was taken to the nearest 0.1 cm.  ***Stool and urine sampling for assessment of gastrointestinal tract infection.*** A sample of at least 15 g of early morning stool from every participant was collected in a container and transferred to a laboratory of the Nelson Mandela University (NMU) in Port Elizabeth for diagnostic work-up. Stool samples were visually examined for Taenia spp. proglottids, signs of blood, mucus, and diarrhea. Duplicate 41.7 mg Kato-Katz thick smears were prepared from each stool sample and examined under a microscope by two experienced laboratory technicians. The number of helminth eggs was counted and recorded for each species separately. Helminth egg counts were multiplied by a factor of 24 to obtain a proxy for infection intensity, expressed as the number of eggs per gram of stool (EPG), which was then categorized into light, moderate, and heavy infections. For the detection of intestinal protozoa C. parvum and G. intestinalis, a Crypto-Giardia Duo-Strip® rapid diagnostic test (RDT) was performed on the stool sample. For the discovery of the bacterium H. pylori, a Pylori-Strip® RDT was applied [22] (both tests are from CORIS, BioConcept; Gembloux, Belgium).  Children were also asked to provide a urine sample, which was transferred to the laboratory and analyzed on the same day. Visual inspection for macrohematuria was followed by testing for blood in urine using Hemastix® strips (Siemens Healthcare Diagnostics GmbH; Eschborn, Germany), as a proxy for *S*chistosoma haematobium infection. A point-of-care circulating cathodic antigen (POC-CCA) urine cassette test (Rapid Medical Diagnostics; Cape Town, South Africa) was used for the diagnosis of Schistosoma mansoni infection [23].  The infectious agents under the same taxonomy were grouped as trematodes (*S. mansoni* and *S. haematobium*), nematodes (*Ascaris lumbricoides*, *Enterobius vermicularis*, and *Trichuris trichiura*), intestinal protozoa (*G. intestinalis* and *C. parvum*), and bacteria (*H. pylori*) in the statistical analyses.  ***HbA1c measurement.***HbA1c reflects plasma glucose concentrations over an 8- to 12-week period. It is used as a convenient diagnostic indicator for DM, as no fasting is required to measure it. HbA1c concentrations were obtained by using the POC instrument Afinion (Alere Inc. Waltham; Waltham, MA, USA), which is based on boronate affinity separation and the use of fluorescence quenching, with results available after 3 min. This method meets the generally accepted performance criteria for HbA1c, as defined by the U.S. National Glycohemoglobin Standardization Program (NGSP), with no interference from HbC, HbS, HbE, and HbD traits results. All test cartridges for the Afinion test belonged to the specific lot number. Test cartridges were stored at 4°C during the study and were removed from the refrigerator a maximum of 120 min before the test. The tests were run when the temperature of the cartridges were in their optimal range (15-25°C). Patients with HbA1c ≥6.5%, the recommended cut-off for diagnosing DM [24], were referred to DM care centers for confirmation and specific management.  ***Hemoglobin (Hb) measurement.*** Hb concentration was measured with the HemoCue® Hb 301 system (HemoCue®AB; Ängelholm, Sweden) and the results were considered to the nearest 0.1 g/l. |
| Bias | 9 | Regression models were *a priori* adjusted for age, sex, SES, Hb, height, weight, BMI, systolic and diastolic blood pressure, physical activity, VO_2_ max, and body temperature to address potential bias. |
| Study size | 10 | The sample size calculation for the study was based on achieving sufficient precision in estimating the prevalence of soil-transmitted helminth infections. We conducted our calculation under the following assumptions for the cross-sectional baseline study: *i)*a prevalence of soil-transmitted helminth infections, p, of approximately 3 %; *ii)*an average number of children per school, B, of 150; and *iii)*an intra-class correlation coefficient for the clustering of outcomes within schools, ICC, of 0.15.  Requiring the standard error of the respective prevalence, SE, not exceeding 2.5 %, we obtained a necessary sample size n of 1088 children, using the formula,  n ⋅≥p⋅(1−p) (1+(B−1)⋅ICC)  SE2  As a consequence, eight clusters (schools) was needed considering the fact, that with a total of 1200 children from eight schools, we can accommodate 10 % loss to follow-up. |
| Quantitative variables | 11 | Descriptive statistics include counts, percentages, means, and standard deviations (SD). The categorization of DM status by sex is described according to the American Diabetes Association cut-offs for HbA1c. The baseline prevalence of the different gastrointestinal tract infections is presented for the different schools separately. The characteristics of covariates at baseline are presented stratified for infected and non-infected children. To assess the association between gastrointestinal tract infections and HbA1c measurement (treated as continuous numerical data) at baseline, linear mixed regressions models with random intercepts for schools were computed. |
| Statistical methods | 12 | (*a*) Models were a priori adjusted for age, sex, SES, Hb, height, weight, BMI, systolic and diastolic blood pressure, physical activity, VO2 max, and body temperature. All models were run (i) by adding each infection separately without excluding children with other infections; (ii) by adding each infection separately and excluding children with other infections; (iii) by adding all infection variables simultaneously and; and (iv) by adding groups of infections. We also assessed dose-response effects on HbA1c for infectious agents, especially *A. lumbricoides* infection, where data on intensity of infection was available. To assess the effect of anthelmintic treatment on changes in HbA1c level between baseline and the 6-month anthelmintic treatment follow-up among children from schools without lifestyle intervention and who were infected at baseline, linear mixed regression models with random intercepts for schools were built. Models were a priori adjusted for age, sex, SES, Hb,height,weight, BMI, physical activity, VO2 max, blood pressure and body temperature, considering information from both time points, as appropriate. Longitudinal models were re-run among subjects infected at baseline but not at follow-up, to differentiate between the effect of the anthelmintic treatment itself and the effect of resolved infection on change in HbA1c. Models were also run for children infected with nematodes and for children with any gastrointestinal tract infection separately. |
|  |  | (*b*) Longitudinal models were re-run among subjects infected at baseline but not at follow-up, to differentiate between the effect of the anthelmintic treatment itself and the effect of resolved infection on change in HbA1c. Models were also run for children infected with nematodes and for children with any gastrointestinal tract infection separately. |
|  |  | (*c*) Complete cases were considered for statistical analysis |
|  |  |  |

Continued on next page

| Results | | |
| --- | --- | --- |
| Participants | 13* | (a) Forty out of 882 participants at baseline moved or changed schools within the 6-month anthelmintic treatment follow-up, and hence, did not participate in the latter cross-sectional survey. |
|  |  | (b) 40 children were not included in the follow up study due to migration or relocation of the children family and the children already left the school. |
|  |  | (c)  1,009 children recruited  882 children remained  842 cases included in analysis  127 cases were ignored due to incomplete data  40 missing children at follow up |
| Descriptive data | 14* | (a) *H. pylori* was the predominant infection (416 children with a positive RDT result, 49.4%). At the unit of the school, the prevalence of *H. pylori* ranged from 27% to 62%.  The second most common infections were the soil-transmitted helminths *A. lumbricoides* and *T. trichiura.* Two out of eight schools showed very high prevalence of *A. lumbricoides* infection (62.5% and 74.1%), there was a moderate infection prevalence in a third school (25.9%), while the prevalence in the five remaining schools were below 5%. High prevalence of *T. trichiura* infection were observed in the same two schools where the prevalence of *A. lumbricoides* prevalence was high (66.7% and 67.9%, respectively), while the prevalence of *T. trichiura* was below 3% in the remaining six schools.  In all schools, infection rates were low to very low or even undetectable for intestinal protozoa (*Cryptosporidium* spp. 1-5%; *G. intestinalis* 6-17%), the nematode *E. vermicularis* (1-5%), and the trematodes *S. mansoni* (1-3%; detected by POC-CCA urine cassette test) and *S. haematobium* (0%).  Except for *H. pylori*, the proportion of children with low SES was higher among infected children compared to their non-infected counterparts. Infections with nematodes and *G. intestinalis* were more common in males, whereas *C. parvum* infection was more common in females. Infected children were, on average, older than their non-infected peers. Nevertheless, children with an *A. lumbricoides*, *T. trichiura*, and *E. vermicularis* infection had lower height, weight, and BMI compared to non-infected children. However, children infected with *A. lumbricoides*, *T. trichiura*, and *H. pylori* reported higher physical activity, but did not differ with regard to cardiorespiratory fitness. Concerning anemia and HbA1c, no clear pattern of association was evident from the univariate analysis. |
|  |  | (b) Forty out of 882 participants at baseline moved or changed schools within the 6-month anthelmintic treatment follow-up, and hence, did not participate in the latter cross-sectional survey. We only consider complete case analysis with the data from 882 participants. |
| Outcome data | 15* | *Cross-sectional study—* At baseline, the overall mean HbA1c level of participants was 5.79% with SD of 0.25. The prevalence of prediabetes and diabetes according to baseline is presented in Supplementary Table S3. 605 (71%) of children had preDM HbA1c levels and three children (0.4%) exhibited HbA1c results ≥6.5% at baseline and were offered diagnostic follow-up for DM. |
| Main results | 16 | (*a*) We observed a positive association between *H. pylori* infection and HbA1c, irrespective of adjustments for other infections (β=0.040; 95% confidence interval (CI) 0.006 - 0.074). No significant association of HbA1c with any other infectious agent or infection group was observed. |
|  |  | (*b*) Results pertaining to the association between albendazole treatment and change in HbA1c level at the 6-month treatment follow-up are presented in Table 4. The analysis is restricted to children with any infection or with nematode infection at baseline, respectively. The regression analyses point to statistically non-significant increases in HbA1c concentrations at the 6-month treatment follow-up. The coefficient was highest in the subgroup of children with any infection at baseline, but no infection at follow-up. |
| Other analyses | 17 | Results pertaining to the association between albendazole treatment and change in HbA1c level at the 6-month treatment follow-up are presented in Table 4. The analysis is restricted to children with any infection or with nematode infection at baseline, respectively. The regression analyses point to statistically non-significant increases in HbA1c concentrations at the 6-month treatment follow-up. The coefficient was highest in the subgroup of children with any infection at baseline, but no infection at follow-up. |
| Discussion | | |
| Key results | 18 | Helicobacter pylori was the predominant infectious agent and showed an independent positive association with HbA1c in a multivariate regression analysis (β=0.040, 95% confidence interval (CI) 0.006-0.073, p<0.05). No association between HbA1c and any other infectious agent was found, but we observed a statistically non-significant increase of mean HbA1c levels after albendazole administration. |
| Limitations | 19 | Reverse causation remains a problem related to the cross-sectional nature of our main analysis. Additionally, the high rate of co-infections may in part mask opposite effects of different parasites on HbA1c. Examining only one stool sample has a low diagnostic accuracy due to the day-to-day and intra-specimen variation in helminth egg output. To partially remedy this shortcoming, test specificity was increased by preparing duplicate Kato-Katz thick smears from each stool sample. Despite the fact that the Alere HbA1c testing is minimally affected by hemoglobinopathies, we cannot assess any influence in the absence of genotyping results. Selection bias related to the complete case analysis approach cannot be excluded but the very high participation rate at baseline and the 6-month anthelmintic treatment follow-up (5% drop-out rate), and the relatively low rate of children not providing stools (15%) are unlikely to have substantially altered the results. |
| Interpretation | 20 | We observed a positive association between *H. pylori* infection and HbA1c, while no statistically significant relationship was observed with any other type of infection. Some animal experiments [27] and human epidemiologic studies [10, 11, 13, 15] have shown helminth infections to lower the blood sugar level and inhibit the development of type 1 DM as well as type 2 DM. An inverse relationship between lymphatic filariasis and both type 1 and type 2 DM was reported from India [10, 11]. Having a previous schistosome infection exhibited a strong protective effect against DM in the People’s Republic of China [12]. *Strongyloides stercoralis* infection seemed to be associated with a reduced risk of type 2 DM in adult Australians [13]. Soil-transmitted helminth infections were linked with an improvement of insulin sensitivity in Indonesia [15]. Diabetic patients in Turkey were found to have a lower prevalence of parasitic disease than their healthy counterparts [16]. In contrast, a positive association was found between *S. stercoralis* infection and DM in Brazil, where it was also found that such infections were associated with a high mortality risk among poorly controlled DM patients [14]. A study conducted by Hakim and colleagues reported a high rate of *G. intestinalis* infection among DM patients [28]. For trematode infections, positive association with HbA1c concentrations were reported from several studies [12, 29, 30]. The cross-sectional nature of these studies precludes casual inference.  *H. pylori* is one of the most common human pathogens causing gastrointestinal inflammation. Potential underlying mechanisms linking H. pylori infection and HbA1c levels and DM may include a disturbance of glucose and lipid absorption by the inflamed gastrointestinal tissue. *H. pylori* infections may also alter host metabolic homeostasis by affecting appetite regulation and energy expenditure through altered balance of ghrelin and leptin secretion, leading to over-eating and metabolic syndrome pathogenesis. The mediating role of gut microbiota alterations remains unknown [31]. The reported associations between H. pylori infection and DM remain inconsistent. The positive association reported among school children in the present study corroborates findings from two large cross-sectional national surveys conducted by Chen and Blaser in American population samples (one aged ≥18 years and one aged ≥3 years) and a Taiwanese study in adults, which all found that *H. pylori* infections were associated with higher mean HbA1c levels [32, 33]. Several smaller outpatient clinic or hospital based studies in Turkey, Pakistan, and Qatar among adults aged 18 years and above showed a higher prevalence of *H. pylori* infection in diabetic patients than non-DM control groups [34-36]. Other studies failed to find a positive association between *H. pylori* and HbA1c or DM [37-39]. In fact, DM patients were found to have higher rates of *H. pylori* eradication therapy according to national health insurance data from Taiwan. *H. pylori* eradication treatment success was found to be lower in DM compared to non-DM patients [40, 41]. Large intervention studies on the long-term impact of *H. pylori* eradication on hyperglycemia and DM development could shed light on the etiologic role of *H. pylori* in DM development given that some studies indicated an improvement of mean HbA1c and insulin resistance in patients with type 2 DM after *H. pylori* treatment [42, 43]. Although not statistically significant, there was an indication in our study for an increase in HbA1c after anthelmintic treatment with albendazole in children harboring nematode infections at baseline. The direction of the effect is in line with the reported shift towards a Th2 response in helminth-infected individuals. A number of clinical trials with helminth or helminth antigen therapy have reported promising results in inflammatory bowel diseases [44], multiple sclerosis [45], rheumatoid arthritis [46], and Chron’s disease [47]. After deworming, which triggers several hyper-inflammatory processes and shifts immune responses from Th2 to Th1, groups of children treated with either albendazole or mebendazole (against soil-transmitted helminthiasis) or praziquantel (against schistosomiasis) had a higher positive response to the skin-prick test and allergy related symptoms [48, 49]. Nevertheless, other studies emphasized that anthelmintic treatment did not have an effect on clinical eczema and asthmatic severity scores [50, 51]. Given that in our study the highest increase in HbA1c after albendazole treatment was observed in children with non-nematode parasite infection, additional research is needed to understand the effect of the anthelmintic drugs on human glucose metabolism. Yet, our results are aligned with the first publication from a randomized placebo-controlled trial in Indonesia, which showed no effect of albendazole treatment on insulin resistance [52]. |
| Generalisability | 21 | The study population exhibited sufficient prevalence range for at least some of the infectious agents investigated to allow for efficient interrogation of the study objective and showed generalizability. |
| Other information | | |
| Funding | 22 | This study was based on the frame of the “Disease, Activity and Schoolchildren’s Health” (DASH) study in Port Elizabeth, South Africa (Yap P, Müller I, Walter C, Seelig H, Gerber M, Steinmann P, et al. Disease, activity and schoolchildren’s health (DASH) in Port Elizabeth, South Africa: a study protocol. BMC Public Health. 2015;15(1):1285. doi: 10.1186/s12889-015-2636-y.). |

*Give information separately for cases and controls in case-control studies and, if applicable, for exposed and unexposed groups in cohort and cross-sectional studies.

**Note:** An Explanation and Elaboration article discusses each checklist item and gives methodological background and published examples of transparent reporting. The STROBE checklist is best used in conjunction with this article (freely available on the Web sites of PLoS Medicine at http://www.plosmedicine.org/, Annals of Internal Medicine at http://www.annals.org/, and Epidemiology at http://www.epidem.com/). Information on the STROBE Initiative is available at www.strobe-statement.org.
