# Supplementary figures and images for: Association between gastrointestinal tract infections and glycated hemoglobin in school children of poor neighborhoods in Port Elizabeth, South Africa
Source: PLoS Negl Trop Dis. 2018 Mar 15;12(3):e0006332. doi: 10.1371/journal.pntd.0006332 (PMC5871004; doi:10.1371/journal.pntd.0006332)

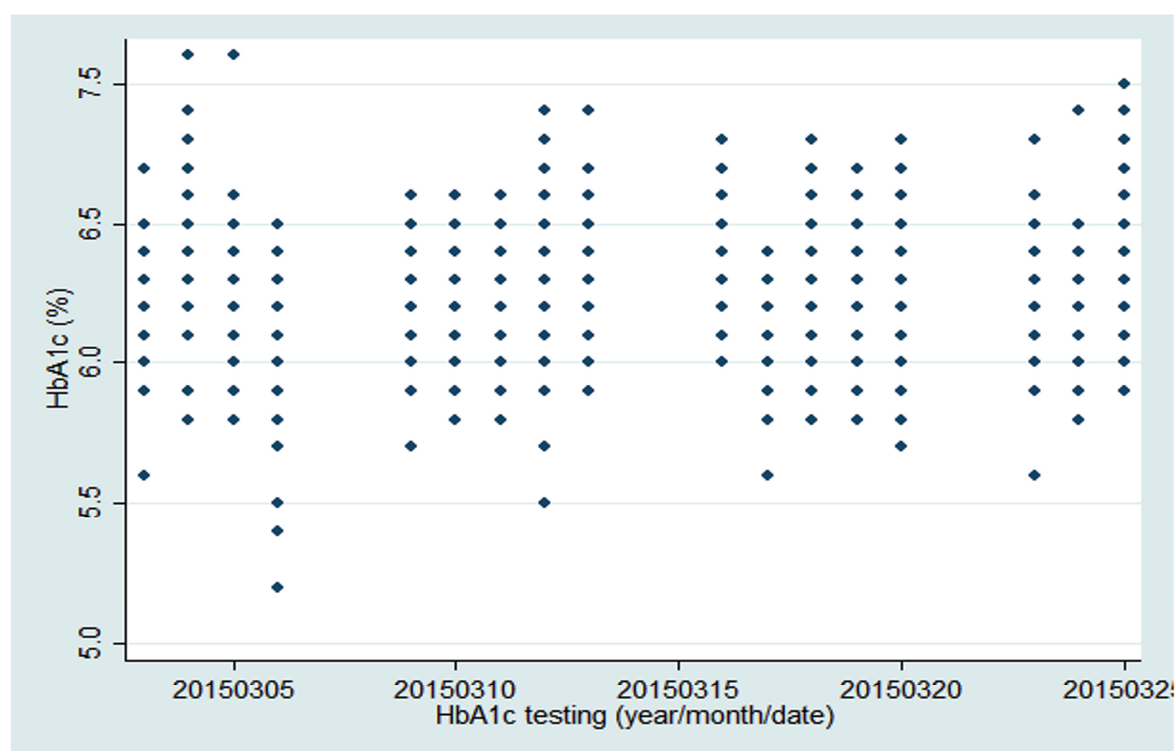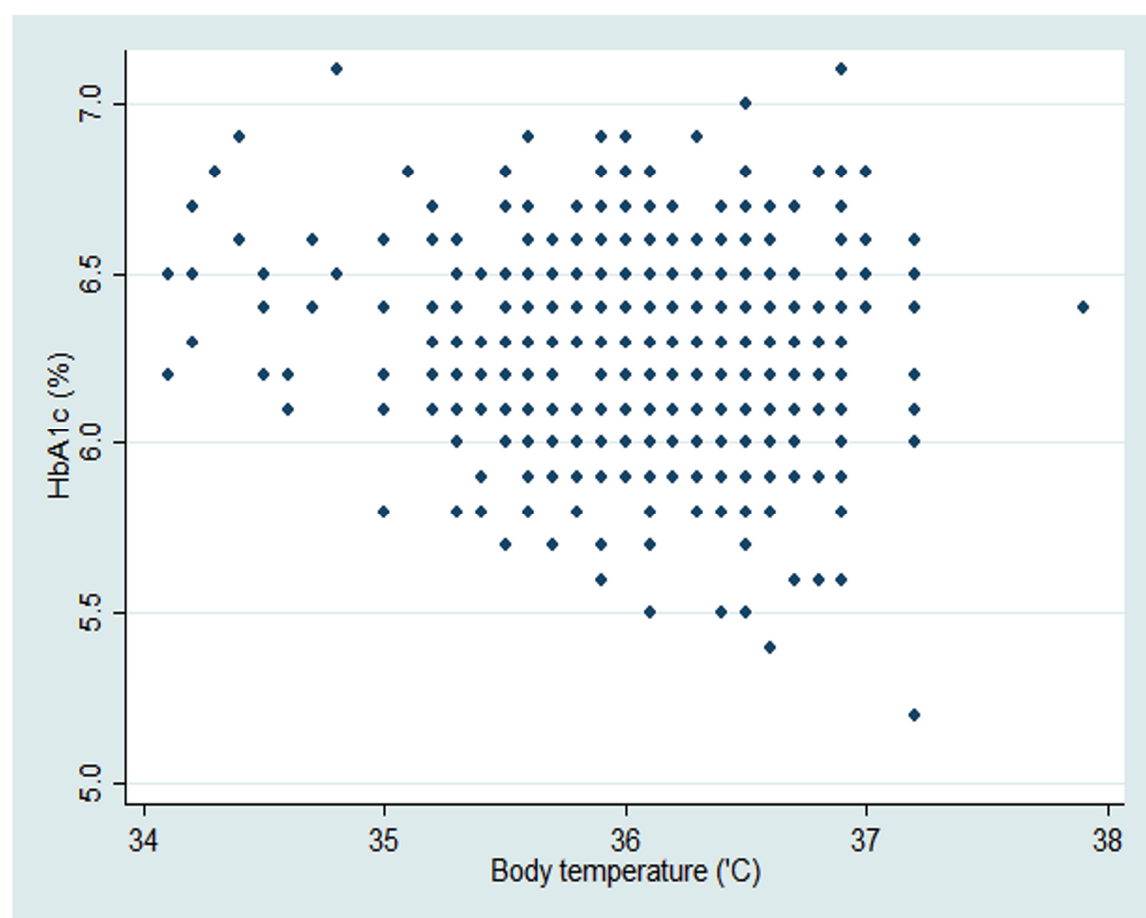

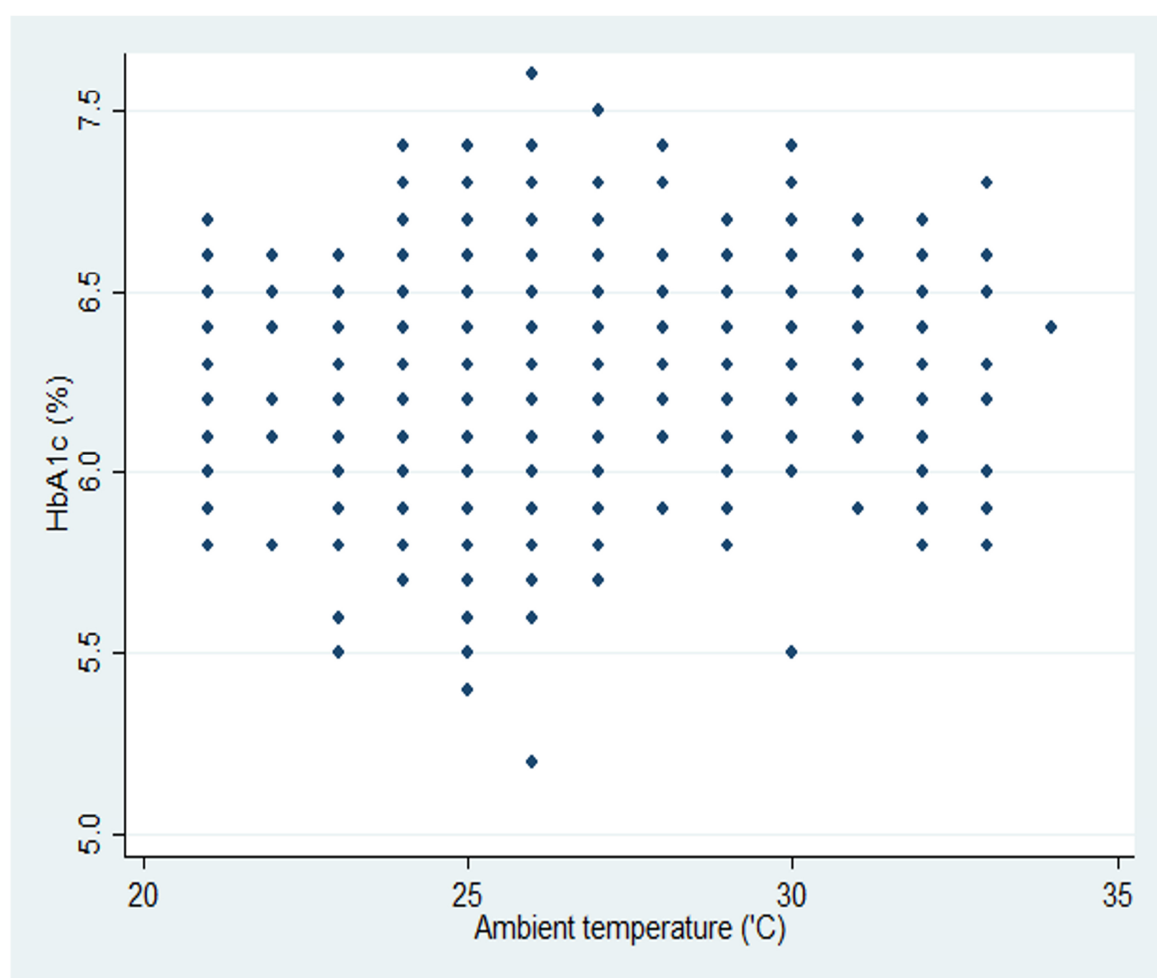

Supplement: S1 Fig — (PDF) [file pntd.0006332.s002.pdf]
