# Supplementary material for: Association between gastrointestinal tract infections and glycated hemoglobin in school children of poor neighborhoods in Port Elizabeth, South Africa
Source: PLoS Negl Trop Dis. 2018 Mar 15;12(3):e0006332. doi: 10.1371/journal.pntd.0006332 (PMC5871004; doi:10.1371/journal.pntd.0006332)
Supplement: S1 Table — (PDF) [file pntd.0006332.s003.pdf]

S1 Table. Multiple Component Analysis (MCA) of SES of the participants

SES variables	Dimension of MCA
**Washing machine**	
Yes	0.571
No	-1.502
**Fridge**	
Yes	0.361
No	-4.047
**Freezer**	
Yes	0.566
No	-2.468
**Radio**	
Yes	0.254
No	-.968
**Landline phone**	
Yes	0.878
No	-0.394
**Television**	
Yes	0.084
No	-2.641
**Car**	
Yes	0.612
No	-0.959
**Computer**	
Yes	0.640
No	-1.013
**Types of houses**	
Shack in informal settlement	-3.735
Backyard shack/room/privately built house	-0.259
RDP/Council house/rented flat/hostel	0.503
**House materials**	
Zinc	0.401
Brick/wood	-0.044
**No of bedrooms in house**	
Only 1 bedroom	-2.447
2 bedrooms	-0.011

> 2 bedrooms	0.884
**Toilet inside the house**	
Yes	0.996
No	-2.007
**Types of toilet**	
Flush toilet	0.562
Pit toilet/bucket/ communal toilet	-3.223
**Types of water sources**	
Taps inside the house	0.820
Taps in the yard/water tank/	-1.719
communal tap/shared with others	-4.098
**Cooking with**	
Electricity	0.209
Gas/Parafin stove/Fire	-3.306

The socioeconomic status (SES) of the children's households was assessed using multiple correspondence analyses (MCA) to be comparable among the schools. The household assets or ownership, the types of the house and construction materials, numbers of bedrooms, water sources, possession and type of latrine, and access to electricity were used to construct a socioeconomic index.

A variable with a positive component score corresponded to higher SES, and conversely, a variable with a negative score to lower SES
