# Supplementary material for: Association between gastrointestinal tract infections and glycated hemoglobin in school children of poor neighborhoods in Port Elizabeth, South Africa
Source: PLoS Negl Trop Dis. 2018 Mar 15;12(3):e0006332. doi: 10.1371/journal.pntd.0006332 (PMC5871004; doi:10.1371/journal.pntd.0006332)
Supplement: S2 Table — (PDF) [file pntd.0006332.s004.pdf]

S2 Table: HbA1c measurements in control probes integrated into baseline and follow up assessment

Controls	Dates (2015)								
	**24/2**	**7/3**	**15/3**	**22/3**	**17/9**	**25/9**	**19/10**	**26/10**	**5/11**
Control 1	6.4	6.4	6.4	6.5	6.6	6.4	6.6	6.5	6.6
Control 2	8.4	8.6	8.7	8.6	8.6	8.4	8.6	8.6	8.6

Normal values of control 1 =5.6-6.8%
Normal values of control 2 =7.3-8.9%
