# Supplementary material for: Association between gastrointestinal tract infections and glycated hemoglobin in school children of poor neighborhoods in Port Elizabeth, South Africa
Source: PLoS Negl Trop Dis. 2018 Mar 15;12(3):e0006332. doi: 10.1371/journal.pntd.0006332 (PMC5871004; doi:10.1371/journal.pntd.0006332)
Supplement: S3 Table — (PDF) [file pntd.0006332.s005.pdf]

S3 Table. Distribution of prediabetes and diabetes based on HbA1c cutoff[1] at baseline, by gender

Categories of HbA1c status	All (N=842) N (%)	Male (N=426) N (%)	Female (N=416) N (%)
Normal	234 (27.8)	105 (25)	129 (31)
Prediabetes	605 (71.8)	319 (75)	286 (68.8)
Diabetes[2]	3 (0.4)	2 (0.5)	1 (0.2)
Mean (Standard Deviation)	5.79 (.250)	5.80 (.251)	5.78 (.250)

[1] The DM status was also categorized into three; normal group: <5.7%, pre DM: 5.7-6.4% and DM: ≥6.5% with the HbA1c results verified by American Diabetes Association [38]

[2] HbA1c values for persons categorized as diabetic were 6.5%; 6.6%; 6.6%.
