# Supplementary material for: Association between gastrointestinal tract infections and glycated hemoglobin in school children of poor neighborhoods in Port Elizabeth, South Africa
Source: PLoS Negl Trop Dis. 2018 Mar 15;12(3):e0006332. doi: 10.1371/journal.pntd.0006332 (PMC5871004; doi:10.1371/journal.pntd.0006332)
Supplement: S4 Table — (PDF) [file pntd.0006332.s006.pdf]

S4 Table. Demographic profile of participants and HbA1c assessment

Characteristic of participants	N	HbA1c Mean	Std Dev
**Age**			
8-9 years	161	5.786	.246
10 years	372	5.776	.250
11 years	215	5.782	.244
12 years	77	5.823	.272
≥12 years	17	5.911	.232
**Ethnicity**			
Black	519	5.829	.242
Indian	2	5.6	.141
Colored	308	5.719	.246
White	5	5.62	.259
Mixed	8	5.863	.381
**Spoken languages**			
Xhosa	513	5.831	.245
Afrikaans	306	5.724	.246
English	21	5.627	.203
Shona	2	6.1	0
Swahili	1	5.8	0
**SES status**			
Poorest	336	5.744	.237
Second poorest	337	5.789	.253
Least poorest	169	5.829	.256
**Housing status**			
Type of housing			
Shack in informal settlement	82	5.793	.244
Backyard shack/room	45	5.748	.217
Privately built house	55	5.741	.237
RDP house	350	5.781	.252
Council house	280	5.815	.256
Rented flat/hostel	30	5.727	.254
**Material of housing**			
Zinc	88	5.826	.274
Bricks	722	5.787	.248
Wood	34	5.7	.231
Others	3	5.667	.057
**No of bedrooms in the house**			
0	5	5.775	.262
1	587	5.718	.226
2	220	5.788	.265
≥3	30	5.841	.239
**Bathroom inside the house**			
No	357	5.754	.248
Yes	485	5.811	.249
**Types of toilet**			
Flush toilet	717	5.796	.252
Pit toilet	12	5.75	.223

Bucket	86	5.757	.242
Communal toilet	27	5.642	.198
**Water sources**			
Taps inside house	630	5.805	.248
Tap in the yard	141	5.708	.252
Water tank	7	5.842	.281
Communal tap/tap shared with others	64	5.773	.234
**Electricity inside the house**			
No	23	5.821	.173
Yes	819	5.786	.252
**Ways of cooking**			
Electricity	792	5.783	.248
Gas	21	5.847	.252
Paraffin stove	28	5.867	.296
Fire	1	5.7	0
**Family related questions**			
Persons who look after the children			
Mother and father	279	5.757	.249
Mother only	252	5.783	.252
Father only	40	5.795	.282
Grandparents	121	5.810	.230
Siblings	92	5.860	.241
Guardians	58	5.769	.260
**Persons with job**			
Both parents/guardians	357	5.777	.249
One parent or one guardian	406	5.786	.248
None	79	5.830	.266
**Government support to the family**			
No	60	5.809	.202
Yes	674	5.780	.255
Dont know	108	5.815	.242
