# Supplementary material for: Association between gastrointestinal tract infections and glycated hemoglobin in school children of poor neighborhoods in Port Elizabeth, South Africa
Source: PLoS Negl Trop Dis. 2018 Mar 15;12(3):e0006332. doi: 10.1371/journal.pntd.0006332 (PMC5871004; doi:10.1371/journal.pntd.0006332)
Supplement: S5 Table — (PDF) [file pntd.0006332.s007.pdf]

S5 Table. Adjusted association of helminth infections with HbA1c at baseline, omitting adjustment for potential mediators and correlated outcomes

Table S5a: Single Infections and infection Groups	All with respective infection[1]			Only respective infection[2]			Mutually adjusted for other infections or groups[3]		
	N	β*	95% CI	N	β*	95% CI	N	β*	95% CI
**Nematodes**	842	-0.012	-0.062 – 0.040	343	-0.036	-0.110 – 0.040	842	-0.020	-0.071 – 0.031
A. lumbricoides	842	-0.013	-0.062 – 0.036	307	-0.039	-0.131 – 0.053	842	-0.022	-0.074 – 0.030
T. trichiura	842	0.008	-0.051 – 0.068	280	0.076	-0.190 – 0.343	842	0.009	-0.155 – 0.073
E. vermicularis	842	-0.055	-0.166 – 0.057	284	-0.078	-0.249 – 0.092	842	-0.058	-0.169 – 0.054
**Trematodes**	842	0.016	-0.055 – 0.087	296	0.014	-0.094 – 0.122	842	0.016	-0.055 – 0.087
S. mansoni	842	0.048	-0.119 – 0.214	278	0.064	-0.388 – 0.516	842	0.042	-0.124 – 0.209
S. haematobium	842	0.009	-0.069 – 0.087	295	0.006	-0.106 – 0.118	842	0.008	-0.070 – 0.086
**Protozoa**	842	-0.003	-0.051 – 0.044	283	-0.011	-0.095 – 0.073	842	-0.003	-0.050 – 0.044
C. parvum	842	-0.009	-0.113– 0.093	283	-0.140	-0.319 – 0.052	842	-0.018	-0.122 – 0.085
G. intestinalis	842	0.005	-0.046 – 0.055	305	0.001	-0.092 – 0.095	842	0.005	-0.046 – 0.059
H. pylori	**842**	**0.038**	**0.004 – 0.071**	488	0.037	-0.006 – 0.080	**842**	**0.040**	**0.007 – 0.073**

Table S5b :Nematode infections	All with respective infection[1]		
	N	β	95% CI
Only nematodes	842	-0.053	-0.121 – 0.015
Nematodes and other infections	842	0.019	-0.039 – 0.078
Only other infections	842	0.012	-0.027 – 0.052

* Beta coefficients reflect the adjusted mean difference HbA1c (%) between children with and without the respective infection. Differences that are statistically significantly different ($p<0.05$) are marked in bold.

[1]Single and group infection models as well as nematode infection models are adjusted schools, for age, sex, socioeconomic status (SES), height, body temperature on the day of the HbA1c test

[2]children with other infections are excluded from this analysis

[3]Mutually adjusted models include either all single infections or all infection groups; H. pylori is included in single infection and infection group models
