# Supplementary material for: Association between gastrointestinal tract infections and glycated hemoglobin in school children of poor neighborhoods in Port Elizabeth, South Africa
Source: PLoS Negl Trop Dis. 2018 Mar 15;12(3):e0006332. doi: 10.1371/journal.pntd.0006332 (PMC5871004; doi:10.1371/journal.pntd.0006332)
Supplement: S6 Table — (PDF) [file pntd.0006332.s008.pdf]

S6 Table. Adjusted association of helminth infections and HbA1c measurement at baseline-DM cases excluded

**S6a table: Single Infections and infection Groups**	**All with respective infection[1]**			**Only respective infection[2]**			**Mutually adjusted for other infections or groups[3]**		
	**N**	**β***	**95% CI**	**N**	**β ***	**95% CI**	**N**	**β***	**95% CI**
**Nematodes**	837	-.020	-.071 .030	341	-.044	-.120 .032	837	-.029	-.080 .022
A. lumbricoides	837	-.022	-.070 .027	305	-.041	-.135 .053	837	-.029	-.080 .023
T. trichiura	837	-.002	-.062 .057	278	.042	-.227 .312	837	.002	-.062 .065
E. vermicularis	837	-.050	-.159 .059	282	-.059	-.230 .113	837	-.053	-.163 .057
**Trematodes**	837	.021	-.049 .091	275	.017	-.092 .125	837	.020	-.050 .090
S. mansoni	837	.043	-.120 .207	276	.088	-.368 .543	837	.040	-.125 .203
S. haematobium	837	.015	-.062 .092	293	.012	-.100 .124	837	.014	-.063 .091
**Protozoa**	837	-.011	-.058 .036	309	-.030	-.115 .054	837	.041	.007 .074
C. parvum	837	-.012	-.113 .090	281	-.123	-.311 .063	837	-.021	-.123 .081
G. intestinalis	837	-.003	-.053 .047	302	-.028	-.122 .065	837	-.002	-.053 .048
H. pylori	**837**	**.039**	**.006 .072**	485	.038	-.005 .082	**837**	**.041**	**.007 .074**

**S6b Table :Nematode infections**	**All with respective infection[1]**		
	**N**	**β**	**95% CI**
Only nematodes	837	-.060	-.127 .007
Nematodes and other infections	837	.009	-.049 .066
Only other infections	837	.012	-.027 .051

* Beta coefficients reflect the adjusted mean difference HbA1c (%) between children with and without the respective infection. Differences that are statistically significantly different ($p<0.05$) are marked in bold.
[1]Single and group infection models as well as nematode infection models are adjusted schools, for age, sex, socioeconomic status (SES), height, body temperature on the day of the HbA1c test
[2]children with other infections are excluded from this analysis
[3]Mutually adjusted models include either all single infections or all infection groups; *H. pylori* is included in single infection and infection group models
