# Supplementary material for: Association between gastrointestinal tract infections and glycated hemoglobin in school children of poor neighborhoods in Port Elizabeth, South Africa
Source: PLoS Negl Trop Dis. 2018 Mar 15;12(3):e0006332. doi: 10.1371/journal.pntd.0006332 (PMC5871004; doi:10.1371/journal.pntd.0006332)
Supplement: S7 Table — (PDF) [file pntd.0006332.s009.pdf]

S6 Table. Adjusted association of *A. lumbricoides* and *T. trichiura* infection intensities and HbA1c measurement at baseline

Infections categories	All with respective infection[1]				Only respective infection				Mutually adjusted for other infections or groups[2]			
	N	β	95% CI		N	β	95% CI		N	β	95% CI	
A. lumbricoides												
No infection	Reference category											
Mild	837	-.057	-.129	.014	305	-.037	-.182	.107	837	-.059	-.132	.013
Moderate	837	-.013	-.071	.045	305	-.029	-.163	.105	837	-.012	-.072	.048
Heavy	837	.011	-.068	.090	305	-.073	-.261	.115	837	.011	-.074	.097
T. trichiura												
No infection	Reference category											
Mild	837	.002	-.063	.067	278	.042	-.227	.317	837	.009	-.058	.076
Moderate	837	-.014	-.089	.062	278	NA	NA		837	-.020	-.100	.060
Heavy	837	.061	-.109	.231	278	NA	NA		837	.056	-.121	.233

[1]Single infection model with adjusted for age, gender, socioeconomic status, schools, hemoglobin level, height, BMI, blood pressure, physical activity, physical fitness, body temperature on the day of the HbA1c test

[2]Mutually adjusted models include either all single infections or all infection groups plus; *H. pylori* is included in single infection and infection group models
