# Supplementary material for: Association between gastrointestinal tract infections and glycated hemoglobin in school children of poor neighborhoods in Port Elizabeth, South Africa
Source: PLoS Negl Trop Dis. 2018 Mar 15;12(3):e0006332. doi: 10.1371/journal.pntd.0006332 (PMC5871004; doi:10.1371/journal.pntd.0006332)
Supplement: S8 Table — (DOCX) [file pntd.0006332.s010.docx]

**S8 Table. Adjusted^1^ estimate of average change in HbA1c (follow-up minus baseline) among children infected at baseline and visiting schools without lifestyle intervention, omitting adjustment for potential mediators and correlated outcomes**

| **Infections exposures** | **N** | **Estimate average change in HbA1c (%)** | **95% CI** |
| --- | --- | --- | --- |
| **Nematode infections** | | | |
| All subjects with a nematode infection at baseline, adjusted for the presence of infection of any type at baseline and follow-up | 414 | 0.039 | -0.027 – 0.104 |
| Subjects with a nematode infection at baseline, but without any infection at follow-up | 217 | -0.016 | -0.114 – 0.080 |
| **Any infection** | | | |
| All subjects with any infection at baseline, adjusted for the presence of infection at follow-up | 260 | 0.063 | -0.012 – 0.138 |
| Subjects with any infection at baseline, but without any infection at follow-up | 103 | 0.022 | -0.082 – 0.126 |

^1^All models were adjusted for schools, age, sex, socioeconomic status (SES), height, body temperature at baseline and follow-up
